# *Hop* Mice Display Synchronous Hindlimb Locomotion and a Ventrally Fused Lumbar Spinal Cord Caused by a Point Mutation in *Ttc26*

**DOI:** 10.1523/ENEURO.0518-21.2022

**Published:** 2022-03-14

**Authors:** Nadine Bernhardt, Fatima Memic, Anna Velica, Michelle A. Tran, Jennifer Vieillard, Shumaila Sayyab, Taha Chersa, Leif Andersson, Patrick J. Whelan, Henrik Boije, Klas Kullander

**Affiliations:** 1 Department of Neuroscience, Uppsala University, Uppsala 751 24, Sweden; 2 Department of Psychiatry and Psychotherapy, University Hospital Carl Gustav Carus, Technische Universität Dresden, Dresden 01307, Germany; 3 Hotchkiss Brain Institute, Department of Comparative Biology and Experimental Medicine, University of Calgary, Calgary, Alberta T2N 4N1, Canada; 4 Department of Medical Biochemistry and Microbiology, Uppsala University, Uppsala 75123, Sweden; 5 Department of Animal Breeding and Genetics, Swedish University of Agricultural Sciences, Uppsala 75007, Sweden; 6 Department of Veterinary Integrative Biosciences, Texas A&M University, College Station, TX, 77843

**Keywords:** central pattern generator, midline fusion, rabbit-like gait, sonic hedgehog, spinal cord, synchrony

## Abstract

Identifying the spinal circuits controlling locomotion is critical for unravelling the mechanisms controlling the production of gaits. Development of the circuits governing left-right coordination relies on axon guidance molecules such as ephrins and netrins. To date, no other class of proteins have been shown to play a role during this process. Here, we have analyzed *hop* mice, which walk with a characteristic hopping gait using their hindlimbs in synchrony. Fictive locomotion experiments suggest that a local defect in the ventral spinal cord contributes to the aberrant locomotor phenotype. *Hop* mutant spinal cords had severe morphologic defects, including the absence of the ventral midline and a poorly defined border between white and gray matter. The *hop* mice represent the first model where, exclusively found in the lumbar domain, the left and right components of the central pattern generators (CPGs) are fused with a synchronous hindlimb gait as a functional consequence. These defects were associated with abnormal developmental processes, including a misplaced notochord and reduced induction of ventral progenitor domains. Whereas the underlying mutation in *hop* mice has been suggested to lie within the *Ttc26* gene, other genes in close vicinity have been associated with gait defects. Mouse embryos carrying a CRISPR replicated point mutation within *Ttc26* displayed an identical morphologic phenotype. Thus, our data suggest that the assembly of the lumbar CPG network is dependent on fully functional TTC26 protein.

## Significance Statement

Our work reveals novel developmental defects in hop mice affecting nervous system development and the assembly of local locomotor circuits. The hop mouse mutant appeared spontaneously in the 1960s, but the underlying cause of the unnatural synchronously hopping gate has not yet been revealed. Altered functionality in hop mutant mice origins from an early developmental defect in the lumbar spinal cord, resulting in a fused ventral midline. The hop mouse represents an animal model harboring a very particular locomotor style that can help us understand the assembly and properties of locomotor networks and how they function to coordinate motor behavior.

## Introduction

In the early 20th century, Thomas Graham-Brown demonstrated that the spinal cord could generate the basic pattern for stepping without descending or peripheral input (Graham-Brown, 1911). Within his model of spinal locomotor control, he implemented Sherrington’s term “half-center” for two groups of reciprocally organized neurons mutually inhibiting each other to provide a coordinated pattern for stepping. Such local spinal networks, capable of generating rhythmic motor output, are referred to as central pattern generators (CPGs). Studies on the spinal cord locomotor CPG have identified key principles for the development and function of neuronal circuitry. For example, mouse mutants with abnormal locomotor coordination have been informative for understanding CPG organization and function (Kullander et al., 2001, 2003; Lanuza et al., 2004; Akay et al., 2006; Lundfald et al., 2007; Crone et al., 2008, 2009; Zhang et al., 2008; Rabe et al., 2009; Restrepo et al., 2009; Zagoraiou et al., 2009; Andersson et al., 2012; Talpalar et al., 2013; Lemieux et al., 2016; Thiry et al., 2018).

The CPGs controlling hindlimb muscle activity during locomotion are located in the ventral part of the lumbar spinal cord (Bracci et al., 1996; Kjaerulff and Kiehn, 1996) whereas CPGs controlling forelimb activity are located in the cervical spinal cord. Inhibitory actions between CPGs on different rostro-caudal levels of the spinal cord coordinate ipsilateral flexor and extensor muscles. Each side of the midline contains CPGs capable of rhythm generation where commissural inhibitory and excitatory projections ensure left-right coordination (Bannatyne et al., 2003; Butt and Kiehn, 2003; Hammar et al., 2004; Lanuza et al., 2004; Quinlan and Kiehn, 2007; Jankowska, 2008; Talpalar et al., 2013). Left-right activity persists when the dorsal spinal cord is removed, whereas left-right alternating fictive locomotion degrades after cutting the spinal cord ventral commissure. These experiments illustrate that ventromedial commissural interneurons (CINs) are critical for bilateral coordination (Kjaerulff and Kiehn, 1996; Restrepo et al., 2009; Andersson et al., 2012).

Neuronal connections form during embryonic development when differentiating neurons send their axons, navigating through the embryonic environment to synaptic targets. This process is mediated by conserved families of axon guidance proteins including netrins, ephrins, slits, and semaphorins (Vallstedt and Kullander, 2013) and impaired axon guidance in the spinal cord results in locomotor coordination dysfunction (Kullander et al., 2001, 2003; Wegmeyer et al., 2007; Rabe et al., 2009; Rabe Bernhardt et al., 2012; Satoh et al., 2016).

In the present study, we analyzed *hop* mice that walk with a characteristic hopping gait using their hindlimbs in synchrony. Mice carrying the hop-sterile (*hop* or hydrocephalic-polydactyly, *hpy)* mutation appeared spontaneously in 1967 in the C57BL/10J strain, and the mutation was first found to be localized at the proximal end of chromosome 6 (Johnson and Hunt, 1971; Lyon, 1973; Hollander, 1976; Bryan et al., 1977). Later a point mutation within the *Ttc26* gene was identified and suggested to be responsible for impaired hedgehog signaling (Swiderski et al., 2014). However, since other genes in the close vicinity have been associated with gait defects, it remained unclear whether the point mutation is causative for the *hop* locomotor phenotype. Using CRISPR technology, we introduced a single point mutation within the *Ttc26* gene, which reproduced the anatomic phenotype observed in *hop* mice. Further, we show that the synchronous gait in *hop* mice is the result of a left and right CPG component fusion exclusively within the lumbar domain of the spinal cord. This is in turn caused by abnormal notochordal and ventral spinal cord signaling during development, resulting in migration defects and an absent ventral midline.

## Materials and Methods

### Animals

#### Mice

CByJ.Cg-hop/J mice were imported from The Jackson Laboratory (N12 on a backcross-intercross to BALB/cByJ). The colony was kept on the BALB/cByJ background for analysis of the locomotor phenotype and outcrossed two to four generations to the C57BL/6J background for positional cloning. *Ttc26^Y430X^* mutant mice were produced in the C57BL/6J background by Siu-Pok Yee at the Gene Targeting and Transgenic Facility, Uconn Health Center. All experiments involving animals were approved by the appropriate local Swedish ethical committee (C147/7 or C79/9), and by the University of Calgary Health Sciences Animal Care Committee.

### Gait study

Two- to three-month-old animals were trained to walk on a 1-m-long and 10-cm-wide track the day before the experiment. Mice were taken up and painting of the paws was mimicked with a brush. Each mouse had to go to the end of the track at least three times without stopping. The day of the experiment mice were handled exactly the same as during the training but this time the paws were painted with two distinct colors for the forelimbs (red) and hindlimbs (blue). Footprints were recorded on Whatmann paper placed at the bottom of the track. For each animal, three runs were recorded and analyzed according to (Kullander et al., 2001).

### Electrophysiology

Experiments were performed on 0- to 3-d-old (Postnatal day (P) 0–P3, weight 1.16–4.05 g; *n* = 62) *hop* homozygote, heterozygote, and wild-type control mice. Mutant mice were identified by preaxial polydactyly of the hindfeet (Extended Data Fig. 1-1) and the synchronous activity in their hindlimbs when suspended in the air with their tails lightly pinched. There were no obvious deficits in gait in the *hop* heterozygote and wild-type control mice.

**Figure 1.**
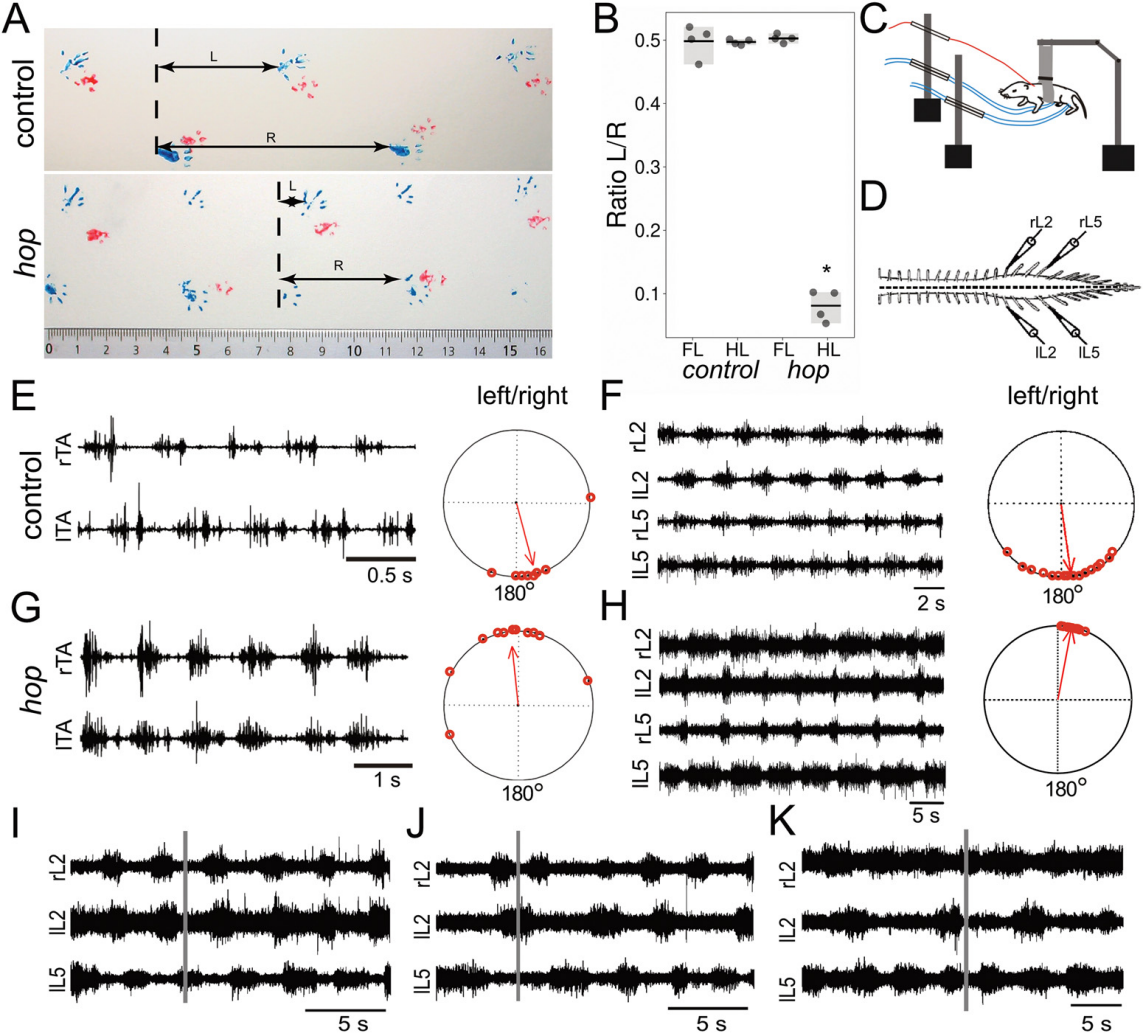
Abnormal gait in hind limbs of *hop* mice. ***A***, Gait analysis painting forelimbs (red) and hind limbs (blue) show that control mice move their forelimbs and hindlimbs in an alternating pattern while *hop* mice move their hind limbs in a synchronous manner. Horizontal arrow L is the distance between the left and right paw and R the distance covered by the same right paw. ***B***, Graph showing quantification of walking pattern with the ratio L/R and significant genotype differences for hindlimb (*p *=* *0.021, *r* = −0.81) but not forelimb (*p *=* *1, *r *=* *0) coordination (*n* = 4 mice per group over a distance of 30 cm). ***C–E***, *Hop* mice show a characteristic synchronous left-right air-stepping pattern *in vivo*. ***C***, Schematic drawing of the experimental setup for recording from neonatal mice. ***E***, ***G***, Raw EMG recordings from the left and right tibialis muscles of *hop* (***G***) and control (***E***) mice following a tail pinch stimulus illustrating representative synchronous and alternating traces, respectively (*n* = 2 mice per group). The circular plots in ***E***, ***G*** represent the phase of onset of the left and right tibialis anterior bursts with respect to each other. Each dot represents an individual burst, while the arrow indicates the tendency for the bursts to be coupled to each other. A phase value close to zero indicates synchrony in the case of the *hop* while a value close to 180° suggests an alternating pattern in the case of the control animals (*p* < 0.01). ***D***, Schematic illustration of ventral root recording setup measuring fictive locomotion. ***F***, ***H***, The *hop* deficit can be reproduced *in vitro* using isolated spinal cord preparations. Control mice exhibit an alternating (***F***) and *hop* mice a synchronous (***H***) left-right pattern (*n* = 20 mice per group). Circular plots (***F***, ***H***) indicate phase values across experiments. Each point equals one 60-s section of data (5 per animal). The length of the arrow provides an index of the strength of the coupling between the rhythms and the direction equals phase. ***I–K***, Bath application of GABA and glycinergic reuptake blockers affect the synchronous rhythm’s stability in the hop mutant mouse. Control rhythm in *hop* mice (***I***) evoked by bath application of NMDA, 5-HT, and DA. Twenty minutes after adding nipecotic acid (***J***), one L_2_ root would double burst occasionally and at random. After application of nipecotic acid and sarcosine, occasional alternating segmental activity could be observed (***K***), although the ipsilateral L_2_–L_5_ rhythm was disrupted. For burst pattern following hemisection, please refer to Extended Data Figure 1-1. *Significant effect of genotype *p *<* *0.05.

10.1523/ENEURO.0518-21.2022.f1-1 Extended Data Figure 1-1 Mid-sagittal hemisection does not disrupt the ipsilateral alternating burst pattern. ***A***, Neurograms illustrating a typical pattern recorded from a *hop* mouse. ***B***, Pattern recorded from the preparation following a midsagittal hemisection. Note the preservation of the L_3_–L_5_ pattern suggesting that flexor-extensor coordination is maintained. ***C***, Schematic illustrating stimulation and recording arrangement for cauda equina stimulation. Cauda equina evoked rhythmic pattern (4 Hz, 10-s train) showed segmental synchronous pattern and ipsilateral alternating pattern. Download Figure 1-1, TIF file.

For *in vivo* experiments, animals were anaesthetized by hypothermia and then suspended in a sling such that the forelimbs and hindlimbs moved freely (Norreel et al., 2003). Electromyographic (EMG) electrodes were inserted into the left and right tibialis anterior muscles parallel to the muscle fibers, and a grounding electrode was inserted subcutaneously into the back. EMG electrodes were made of 75-mm Teflon-coated platinum-iridium wires (AM Systems Inc.). A heat lamp was used to keep the air temperature at 30°C. Recordings were amplified (1000 times), bandpass filtered (100–1 kHz), and digitized at 3 kHz (Axon Digidata 1322A) for future analysis. Five to 10 min after the EMG wires were inserted, air-stepping sequences were elicited by pinching the tail with forceps and the activity was recorded.

For *in vitro* experiments, the animals were anaesthetized by hypothermia. Animals were rapidly decapitated, eviscerated and the remaining tissue was placed in a dissection chamber filled with oxygenated (95% O_2_–5% CO_2_) artificial CSF (ACSF; 128 mm NaCl, 4 mm KCl, 0.1 mm CaCl_2_, 2 mm MgSO_4_, 0.5 mm Na_2_HPO_4,_ 21 mm NaHCO_3_, and 30 mm D-glucose). A ventral laminectomy exposed the cord, and the ventral and dorsal roots were cut. The spinal cord was transected at thoracic 1–3 (T_1–3_) to sacral 2–3 (S_2–3_) and carefully removed from the vertebral column. After 30 min, the preparation was transferred to the recording chamber and superfused with oxygenated ACSF (128 mm NaCl, 4 mm KCl, 1.5 mm CaCl_2_, 1 mm MgSO_4_, 0.5 mm Na_2_HPO_4_, 21 mm NaHCO_3_, and 30 mm D-glucose). The bath solution was then heated gradually from room temperature to 27°C. The preparation was allowed to acclimate for 1 h thereafter. Population motoneuron bursting activity was recorded using suction electrodes into which segmental ventral roots from the left and right lumbar L_2_ and L_5_ segments were drawn (Whelan et al., 2000). In some experiments, we found that the L_2_ ventral root was not well formed, and in these cases, we recorded from the L_3_ ventral root. Neonatal mice show synchrony between L_1_, L_2_, and L_3_ ventral root neurograms (Whelan et al., 2000). The resultant neurograms were amplified (100–20,000 times), band pass filtered (100 Hz to 1 kHz) and digitized at 2–5 kHz (Axon Digidata 1320) for future analysis. Ten minutes of control baseline activity was recorded before adding drugs; 5 μm *N*-methyl-DL-aspartic acid (NMA; Sigma-Aldrich), 10–20 μm serotonin (5-HT; Sigma-Aldrich), and 50–75 μm dopamine (DA; Sigma-Aldrich) were added to the bath and the rhythm stabilized for 10–30 min. In some experiments, a GABA uptake inhibitor (0.1–1 mm nipecotic acid or 50–100 μm NO-711; Sigma-Aldrich) and/or a glycine uptake inhibitor (100 μm sarcosine; Sigma-Aldrich) was added to the bath solution. In some experiments, we examined whether a hemisected spinal cord was capable of generating coordinated rhythmic activity. In these experiments, we first examined the activity patterns in the presence of rhythmogenic drugs. We then removed the recording electrodes and using a pair of micro-clippers completely mid-sagittally hemisected the spinal cord. The suction electrodes were then reattached to the ventral roots.

Data were digitally rectified, integrated and then analyzed using custom written programs (MATLAB, MathWorks). Locomotor-like activity was quantified using time series analysis. Time series analysis was performed by taking intervals of 60 s of raw data, rectifying the data, applying a low-pass filter and resampling at 100 Hz. Means were subtracted from the processed data and further smoothed using a digital filter (Savitzky–Golay, third order polynomial operating over 13 points). Cross and auto-correlograms were then calculated and the quality of the rhythm was assessed by measuring the correlation coefficients for the segmental L_2_ ventral root bursts and the left L_2_ and left L_5_ ventral root bursts. To measure rhythm stability, the peak-to-trough correlation coefficient (PTCC) was calculated from the cross correlogram by subtracting the minimum negative value of the correlation coefficient from the maximum of the first positive peak over the first 75 lags (each lag = 50 ms). Stable synchronous rhythms typically have high positive correlation coefficients at zero phase lag. The cycle periods for the resultant rhythm were calculated by measuring the number of lags from one peak to the next from the auto-correlogram. The phase lag between ventral root bursts was obtained from the cross correlogram and was defined as the distance from the minimum trough around lag 0 to the next peak divided by the cycle period (Madriaga et al., 2004). Data are expressed as mean ± SEM, and significance was analyzed using paired and unpaired Student’s *t* tests if the data were normally distributed (*p* < 0.05). Data that were not normally distributed were analyzed using a Wilcoxon signed-rank test. Multiple data points were analyzed using a one-way ANOVA followed by a Tukey’s *post hoc* test to detect significant differences. To analyze *in vivo* EMG data and to illustrate phase relationships for selected *in vitro* experiments, we used circular statistics, in which the phase was normalized from 0 to 1 (Drew and Doucet, 1991). If the length of the arrow is large this suggests a tendency for the rhythms represented by the two neurograms to be coupled. Significance was computed using Rayleigh’s test (*p* < 0.05).

### Tracing of CINs

Fluorescent dextran-amines 3000 MW rhodamine-dextran-amine (RDA) and 3000 MW fluorescein-dextran-amine (FDA; Invitrogen) were used for retrograde tracing of CINs as described previously (Eide and Glover, 1995). P0–P3 mice spinal cords were prepared as described earlier (Wegmeyer et al., 2007). Tracings on embryonic day (E)12.5 embryos were performed essentially the same way, but the spinal cord remained within the vertebral column during tracer application and incubation. Preparations were incubated for 12–16 h and then fixed in 4% paraformaldehyde (PFA) in 0.1 m PBS, pH 7.4 and stored dark at 4°C for one week. Spinal cords were cut into 60-mm-thick transverse sections on a vibratome (Leica) and stored in the dark at –20°C until analysis. Embryonic tracings were fixed for 2 h, transferred to 30% sucrose in PBS at 4°C over-night, embedded in OCT and 12-mm sections were cut using a cryostat (CM1800, Leica), collected onto Superfrost slides (Menzel-Gläser).

### *In situ* hybridization

Free floating vibratome sections were rehydrated in consecutive washes for 10 min in 75%, 50%, and 25% methanol in PBT, bleached in 6% hydrogen peroxide in PBT for 15 min and treated with 0.5% Triton X-100 for 5 min. The sections were digested with proteinase K (10 μg/ml) in PBT for 15 min. The digestion was stopped with a wash in glycine (Scharlau Chimie; 2 mg/ml) in PBT for 5 min, and sections were postfixed in 4% formaldehyde for 20 min. The sections were prehybridized at 65°C in hybridization buffer [50% formamide, 5xSSC pH 4.5, 1% SDS, 50 μg/ml tRNA (Sigma), and 50 μg/ml heparin (Sigma)] for 2 h before addition of probe; 1 μg/ml probe was added to the hybridization buffer, and sections were hybridized over-night at 65°C. Excess probe was removed by washes in wash buffers (50% formamide, 5xSSC pH 4.5 and 1% SDS; 50% formamide, 2xSSC pH 4.5, and 0.1% Tween 20) at 65°C for 3 times 30 min each. The sections were transferred to blocking solution (1% blocking reagent in TBST) for 2 h before addition of anti-DIG AP (1:5000) diluted in blocking solution and incubated over-night at 4°C. The sections were treated with levamisole (0.5 mg/ml) in TBST and levamisole (0.5 mg/ml) in NTMT (100 mm NaCl, 10 mm Tris-HCl pH 9.5, 50 mm MgCl_2_, and 0.1% Tween 20) before developing in BM purple AP substrate (Roche) at 37°C 1 h to 4 d. Between additions of new chemicals, sections were washed with either PBT (before addition of probe) or TBST (after addition of probe). *In situ* hybridization on cryosections was performed essentially as described (Schaeren-Wiemers and Gerfin-Moser, 1993). The vesicular inhibitory amino acid transporter (VIAAT) probe covers nucleotides 588–2072, the Vglut2 probe 1616–2203 used as described earlier (Wallén-Mackenzie et al., 2006), the vesicular acetylcholine transporter (VAChT) probe 1534–2413, Netrin-1, and EphrinB3 probe 1–1021.

### Immunohistochemistry

The tissue was cryoprotected in 30% sucrose in PBS, embedded in OCT medium and sectioned at a thickness of 12–14 μm on a cryostat (CM1800, Leica). The sections were washed in PBS and preblocked in blocking solution, 5% goat serum, 0.3% bovine serum albumin (BSA; Sigma-Aldrich), and 0.1% Triton X-100 (Sigma-Aldrich) in PBS. Primary antibodies were diluted in blocking solution, added to the sections and incubated over night at 4°C. The following dilutions were used: mNkx2.2 1:100 (Hybridoma bank), mEvx1 1:50 (Hybridoma bank), gpLbx1 1:10,000 (kind gift from C. Birchmeyer), rPax2 1:1000 (Covance), mBrn3a 1:500 (Chemicon), mIsl1/2 1:100 (Hybridoma bank), mLhx1/5 1:500 (Hybridoma bank), Lhx2/9 1:8000 (kind gift from T. Jessell), mShh 1:100 (Hybridoma bank), mMap2 1:500 (Chemicon), rbNeuN 1:1000 (Chemicon), myelin basic protein (MBP) 1:500 (Abcam), rbTtc26 1:1000 (Novus Biologicals), mNeuN 1:500 (Chemicon), gpVAChT 1:500 (Millipore), gChAT 1:250 (Millipore), mParvalbumin 1:1000 (Sigma), and gpDmrt3 1:10,000 (custom made using the immunizing peptide CKQSIYTEDDYDERS-amide).

For some antibodies, antigen retrieval was necessary to get good immunohistochemical staining. The sections were then washed with PBS and thereafter placed in a metal rack inside a beaker filled with preheated citrate buffer (9 ml 0.1 m citric acid, 41 ml 0.1 m trisodium citrate dehydrate and 450 ml dH_2_O, pH 6.0). After 30 min at 98°C, the beaker was left to cool down at room temperature for 30 min. Subsequently the sections were washed with dH_2_O for 1 min and preblocked in 1% normal goat serum and 0.1% Triton X-100 in PBS for 1 h. Finally, the primary antibodies were diluted in blocking solution (1% normal goat serum and 0.1% Triton X-100 in PBS), added to the sections and incubated over night at 4°C. The following day, the sections were washed with PBS and incubated with DAPI (Sigma-Aldrich, 200 ng/ml) and the secondary antibodies (diluted 1:500 in blocking solution) for 2–3 h at room temperature. After washing with PBS, the sections were mounted in Mowiol 4-88 (Sigma-Aldrich). Secondary antibodies used: goat anti-mouse conjugated with FITC 488 (F(ab’)_2_Fragment, 111-096-003, Jackson ImmunoResearch), donkey anti-guinea pig conjugated with Alexa Fluor 594 (Jackson ImmunoResearch), donkey anti-rabbit conjugated with Alexa Fluor 488 (Invitrogen) and donkey anti-goat conjugated with Alexa Fluor 488 (Jackson ImmunoResearch).

*Ttc26^Y430X^* mice embryos (E16.5) were euthanized, genotyped, fixed in 4% PFA for 4 h on a rocking table at room temperature and then washed in 0.1 m PBS (Sigma-Aldrich) for 30 min before being cryoprotected in 30% sucrose in PBS. The tissue was shipped in 30% sucrose in PBS (in a falcon tube placed inside a Styrofoam box with cool-packs) to Uppsala University. Upon arrival 5 d later, the embryos were frozen in OCT medium (Richard Allan Scientific NEG50) on dry ice. Cryostat sections were cut (cryostat CM1800, Leica) at a thickness of 14 μm and placed on Superfrost slides (Menzel-Gläser). For the immunohistochemistry experiment, postnatal P2 wild-type rabbits from the Sauteur d’Alfort breed were obtained from the Fédération Française de Cuniculiculture. They were anesthetized with a mix of medetomidine (Dormilan 0.1 ml/kg) and ketamine (Ketamidor 0.15 ml/kg) and cardiac perfusion was performed with PBS followed by formaldehyde 4% in PBS. The spinal cords were dissected and postfixed overnight at 4°C in formaldehyde 4% in PBS.

### Histology staining

For hematoxylin and Eosin staining, cryosections of spinal cord were rehydrated in dH_2_O for 1 min, stained with progressive hematoxylin (Mayer’s) for 90 s, washed twice in dH_2_O for 1 min, dipped in Scott’s tap water (20 g MgSO_4_·7H_2_O and 2 g NaHCO_3_ in 1-l tap water), dehydrated in 96% ethanol in dH_2_O for 1 min, and finally counterstained with 0.5% eosin (Sigma-Aldrich) in dH_2_O for 20 s. Afterwards the sections were dehydrated with successive washes in 70% and 96% ethanol in dH_2_O for 1 min and treated with X-tra Solve(MEDITE) for 4 min before being mounted in Pertex (Histolab). 3,3′-diaminobenzidinetetrahydrochloride (DAB; Sigma) was used on free-floating spinal cord sections to visualize white matter (dark brown) and Luxol fast blue (LFB) was used to analyze paraffin embedded brain sections staining myelin and phospholipids (blue-green) counterstained with cresyl violet as previously described (Kullander et al., 2001).

For the histology experiment, wild adult rabbits were euthanized in Stockholm by Stockholms Vilt- och Skadedjursgrupp and rabbit tissue was shipped on ice to Uppsala University within a few hours. Upon arrival, the vertebral column was placed in 4% PFA in PBS for two weeks, before the spinal cord was dissected. Thereafter the tissue was cryoprotected in 30% sucrose in PBS for 60 h and frozen in OCT medium (Richard Allan Scientific NEG50TM) on dry ice.

### CRISPR mutagenesis

Based on previously published data (Swiderski et al., 2014) and our independent exome sequencing validation (Extended Data Fig. 7-1), a point mutation located at Chr6:38 362 071 (C>A) and resulting in a nonsense mutation (Y430X) within the *Tetratricopeptide repeat protein 26* (*Ttc26*) gene was identified in *hop* mice. We used CRISPR/Cas9-mediated genome editing technology with the help from Siu-Pok Yee, Ph.D. at the Gene Targeting and Transgenic Facility, Uconn Health Center. A C/A transversion at position 38412065 in exon 15 of the gene *Ttc26* on chromosome 6 was created (Inui et al., 2014) to replace a TAC codon in the amino acid chain for tyrosine 430 with a TAA to create a premature STOP codon. A silent point mutation was also introduced to create a novel PvuII site, 3′ of the knock-in termination codon in *Ttc26* to allow for straightforward genotyping of the mice by PCR followed by a PvuII digestion. The PvuII site also affected the PAM sequence of the CRISPR so re-digestion of the KI allele was avoided. CAS nuclease, a guide RNA and an oligonucleotide carrying the desired mutation and homology arms were co-injected into the nuclei of fertilized oocytes, followed by implantation of the eggs into surrogate mothers to obtain offspring. An allele-specific primer was developed for genotyping of the pups. We obtained one *Ttc26* Y430X founder female that appeared to be heterozygous for the mutation (1:1 peak ratio from sequencing). From F1 pups from the founder female, we bred heterozygous males and females to obtain homozygous *Ttc26^Y430X^* mouse mutants.

### Imaging and picture processing

Fluorescent and bright field images were acquired either on an Olympus BX61WI microscope (Olympus) using the Volocity software (Improvision) or on a MZ16F dissection microscope with a DFC300FX camera and FireCam software (Leica). Close-ups were taken using the OptiGrid Grid Scan Confocal Unit (Qioptiq) or a confocal microscope (Zeiss LSM 510 Meta). For illustrations, captured images were auto-leveled using Adobe Photoshop software. Quantitative image analysis was performed using the Fiji processing suite (REF). For each image, regions of interest (ROIs) were manually defined. For quantification of fluorescence, a global threshold was applied before relevant object information was extracted. Distribution plots were obtained using R Studio version 4.0.4 (R Core Team, 2021) and the spatstat package (Baddeley and Turner, 2005).

### Multiple sequence alignments of TTC26

An amino acid sequence analysis of TTC26 was performed to generate multiple sequence alignment. In order to perform this analysis, we first downloaded the protein sequences from the National Center for Biotechnology Information (NCBI) for multiple species (human, rabbit, dog, cow, pig, chimpanzee, mouse, and rat) with the accession number (NP_079202.2, XP_002722121.2, XP_003639595.2, XP_002687045.1, XP_003134668.2, XP_527905.3, NP_705828.2, NP_001020216.1) respectively. Multiple sequence alignments were performed using MUSCLE software (Edgar 2004, PMID: 15034147) with default parameters, in the multiple sequence alignment editor SEAView (Galtier 1996, PMID: 9021275) version 3.2 by setting the human sequence as the reference with the block size of 10.

### Statistical analysis

Simple effects of genotype (crtl, *hop*) were analyzed using independent *t* test (two-tailed) or Mann–Whitney *U* test dependent on data distribution. Analyses were performed using MS Excel 2016 or SPSS Statistics (version 27.0, IBM). Data were plotted using R (https://www.r-project.org) and the package shiny and vioplot. Statistical significance was set at *p* < 0.05, effect sizes are reported with Cohen’s *d* or *r* (rank-biserial correlation) for nonparametric testing.

## Results

### Aberrant synchronous hindlimb coordination in *hop* mice correlates to local spinal cord neuronal circuitry

Homozygous *hop* mutants show distinct phenotypes such as preaxial polydactyly of all feet, sperm tail deficiency resulting in male sterility, and hydrocephalus (Johnson and Hunt, 1971; Hollander, 1976; Swiderski et al., 2014). Although there have been no detailed reports on the locomotor pattern produced by *hop* mutant animals, it has been described that they exhibit a characteristic hopping gait (Johnson and Hunt, 1971; Wojnowski et al., 1998). We confirmed and extended these observations by conducting gait analysis and electrophysiological experiments. Gait analysis in adults revealed that during locomotion, mice homozygous for the *hop* mutation, herein referred to as *hop* mice, alternated their forelimbs while their hindlimbs moved in synchrony (Fig. 1*A*,*B*). To assess the onset of this hopping pattern, we evoked locomotor activity by pinching the tail, followed by EMG recordings from the *tibialis anterior* muscles in conscious early postnatal (P0–P5) *hop* and control animals. We found that *hop* mice produced an air locomotor pattern of synchronous hindlimb movements. As expected, control neonatal mice produced a typical left-right alternating pattern (Fig. 1*C–E*).

To determine whether deficiencies in the spinal cord CPG are the sole cause of the locomotor phenotype, we performed experiments using isolated spinal cord preparations. Following bath application of 5-HT, DA, and NMDA, we found that in the majority of *hop* mutants (20/28), a synchronous fictive locomotor rhythm developed between left-right ventral roots. In contrast, the alternating ipsilateral flexion-extension like pattern between the L_2_ and L_5_ roots was preserved (Fig. 1*F–H*). We observed that in contrast to control mice the L_5_ burst duration was extended and an asymmetry was observed between the L_2_ and L_5_ burst duration in 13/20 *hop* mutants. The data represent an average for 5 min following the development of a sustained rhythm [L_2_–L_2_: 1.10 ± 0.06 (PTCC), 5.61 ± 0.68 s (cycle period), 0.05 ± 0.01 (phase lag); L_2_–L_5_: −1.04 ± 0.06 (PTCC), 0.52 ± 0.03 (phase lag), *n* = 20]. Although the dominant rhythm observed was a synchronous left-right and an ipsilateral alternating pattern, it was not the only one. 5/28 *hop* mutant mice exhibited a left-right alternating pattern consistent with a normal locomotor-like pattern. In 3/28 *hop* mutant mice, we observed a synchronous pattern between all roots [L_2_–L_2_: 1.26 ± 0.10 (PTCC), 11.78 ± 3.76 s (cycle period), 0.01 ± 0.01 (phase lag); L_2_–L_5_: 1.14 ± 0.13 (PTCC), 0.01 ± 0.003 (phase lag)]. Moreover, the data indicate that the fictive locomotion in *hop* mice was less stable than in control mice, and in 5 of the 20 animals that displayed a synchronous segmental left-right and ipsilateral alternating rhythm, it was sustained for around 5 min, which was notably different from control mice, where the standard alternating rhythm was sustained for up to 60 min. There were no significant differences in cycle periods between *hop* mutants and controls [L_2_: 5.19 ± 0.39 s, L_5_: 5.53 ± 0.37 s (mutants); L_2_: 5.83 ± 0.98 s, L_5_: 5.33 ± 0.80 s (controls); *n* = 8; *p* > 0.5]. The synchronous fictive locomotion we observed in 20/28 *hop* mice was in accordance with the *in vivo* gait analyses as well as the EMG recordings.

In the 20 animals that generated synchronous segmental patterns and ipsilateral alternation, we found that the ipsilateral pattern of L_2/3_–L_5_ alternation was maintained following hemisection, suggesting that the circuitry coordinating flexor-extensor alternation was intact in these *hop* mutants (Extended Data Fig. 1-1). Following hemisection, it was usually necessary to increase the concentration of the drug cocktail (DA to 75 μm, 5-HT to 15 μm) to maintain rhythmic activity [L_2/3_–L_5_: 1.18 ± 0.15 (PTCC), 5.53 ± 3.03 s (cycle period), 0.51 ± 0.04 (phase lag), *n* = 2]. The increase in concentration is typical of previous reports (Whelan et al., 2000).

The asymmetry between flexor and extensor bursts was not present in the hemisected cords (Extended Data Fig. 1-1*B*) and was qualitatively similar to reports using normal mice (Whelan et al., 2000).

To further establish whether synchronous left-right rhythmic activity was the dominant pattern, we used electrical stimulation of the cauda equina or dorsal roots to elicit a rhythm (Whelan et al., 2000; Gordon and Whelan, 2006). We found that in *hop* mice, electrical stimulation did evoke a synchronous rhythm similar to that observed using pharmacological stimulation (Extended Data Fig. 1-1*C*). In addition, in several mice, we were able to observe spontaneous bouts of synchronous left-right and alternating L_2_–L_5_ rhythm that would last a few seconds. Collectively, these data support the idea that the *hop* deficit is at least partly because of alterations in the lumbar network.

Previous studies have shown that neurotransmitter balance over the midline is critical for coordinated left-right output of the CPG, and during fictive locomotion, the balance between excitation and inhibition can be shifted using pharmacological strengthening (Kullander et al., 2003; Hinckley et al., 2005; Juvin et al., 2005; Wu et al., 2018). Following administration of nipecotic acid or NO-711 (data pooled since no difference was observed between the two drugs), we found that, although the left-right pattern did not switch to an alternating pattern, the rhythm became less stable [Fig. 1*I*, control: L_2_–L_2_: 1.11 ± 0.06 (PTCC), 6.34 ± 1.56 s (cycle period), 0.03 ± 0.01 (phase lag); L_2_–L_5_: −1.10 ± 0.04 (PTCC), 0.49 ± 0.04 (phase lag); with nipecotic acid or NO-711 (pooled data): L_2_–L_2_: 0.75 ± 0.33 (PTCC), 4.88 ± 0.55 s (cycle period), 0.11 ± 0.06 (phase lag); L_2_–L_5_: −1.01 ± 0.10 (PTCC), 0.73 ± 0.13 (phase lag); *n* = 7]. We also tested both sarcosine and nipecotic acid bath applied simultaneously in a separate series of experiments. We found that this manipulation consistently altered network dynamics by changing the synchronous pattern to a less stable pattern, which at times appeared to be alternating [Fig. 1*I*, right panel; control: L_2_–L_2_: 1.29 ± 0.13 (PTCC), 3.73 ± 0.35 s (cycle period), 0.06 ± 0.003 (phase lag); L_2_–L_5_: −1.17 ± 0.07 (PTCC), 0.58 ± 0.03 (phase lag); with sarcosine and nipecotic acid: L_2_–L_2_: 0.27 ± 0.44 (PTCC), 4.54 ± 0.23 s (cycle period), 0.24 ± 0.15 (phase lag); L_2_–L_5_: −1.05 ± 0.10 (PTCC), 0.55 ± 0.04 (phase lag); *n* = 3].

Taken together, our locomotor analysis of *hop* mice confirmed the synchronous pattern previously reported and that defects in the lumbar spinal cord circuitry are sufficient to explain the altered hindlimb coordination. Further, whereas changing the balance of excitatory and inhibitory connections over the midline in *hop* mice caused altered network activity, the synchronous left-right output was not consistently switched back to alternation.

### Altered spinal cord morphology in *hop* mice

**Figure 2.**
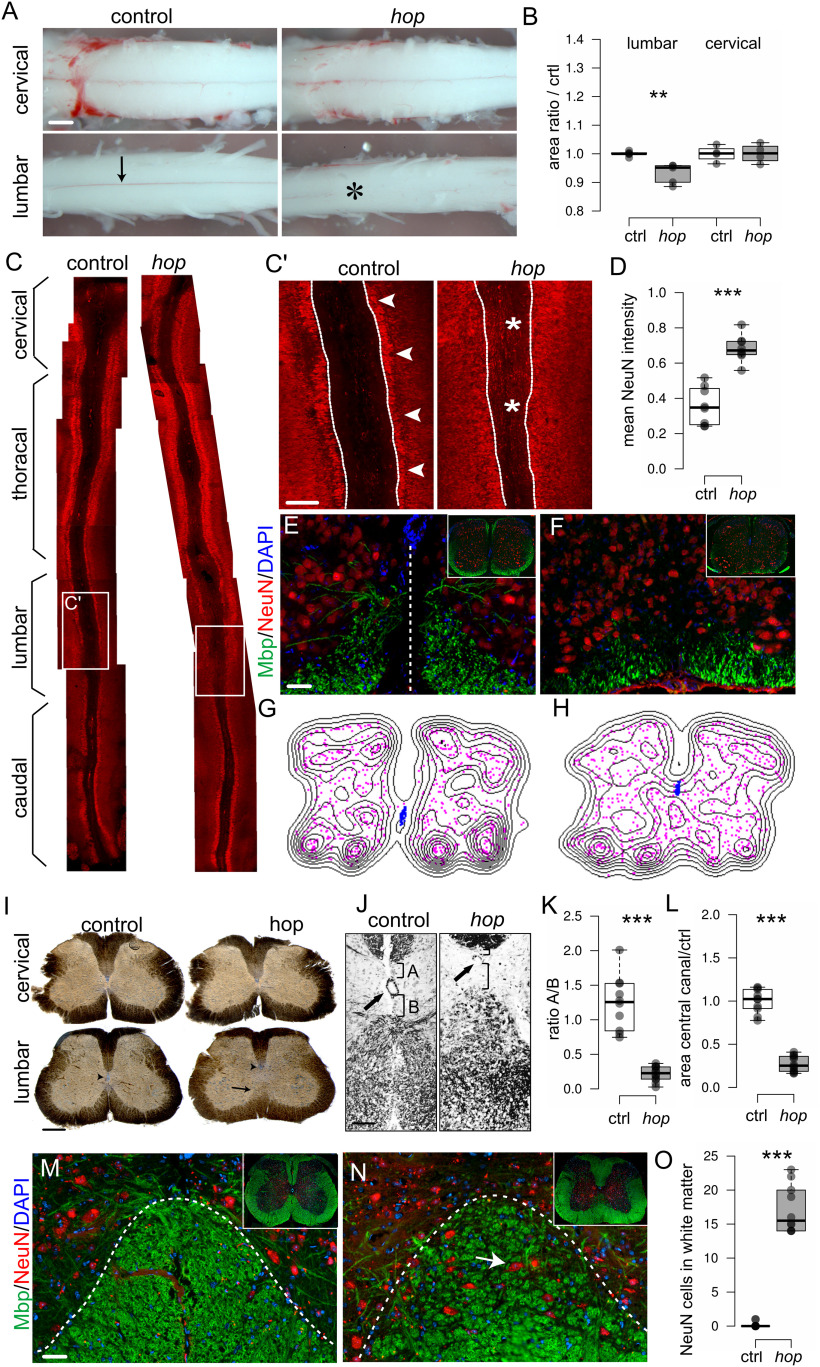
Altered spinal cord morphology in postnatal *hop* mice. ***A***, Photomicrographs (ventral view) and (***B***) graph of measured sizes of early postnatal (p0–p5) cervical and lumbar spinal cords. At cervical levels from control and *hop* mice, no difference is apparent (*p *=* *1, *r *=* *0; *n* = 4 animals per group). At lumbar levels, the lumbar enlargement of the spinal cord is less prominent in *hop* mice (black bar) compared with control mice (white bar, *p *=* *0.009, *r* = −0.83; *n* = 5 animals per group). The visible ventral spinal artery in control wild-type mice (arrow) is absent from the lumbar part of the *hop* spinal cord (position noted with star). ***C***, Photomicrographs of immunohistochemistry staining with NeuN in red on spinal cord open-book preparations demonstrated lumbar specific defects in *hop* mice. White boxes indicate areas of higher magnification displayed in ***C'***. ***C'***, Arrows point to the border between white matter and NeuN**-**positive cells, and stars denote areas where aberrant NeuN**-**positive cells are found. ***D***, Fluorescence intensity analysis of NeuN signal within ROI as indicated (dotted lines in ***C'***) reflects aberrant NeuN positioning in *hop* mice (*p *=* *0.009, *r* = −0.83; *n* = 8 per group). ***E–H***, Immunohistochemistry staining with MBP in green and NeuN in red, coupled to density plots in ***G***, ***H***, shows misplaced neurons in the ventral spinal cord and a dorsally shifted central canal in *hop* mice at P5 (***F***, ***H***) compared with control (***E***, ***G***). ***I***, Peroxidase staining on 60-μm transverse sections of adult spinal cords showed abnormal morphology in the lumbar spinal cord of *hop* mice. The central canal is dislocated dorsally and is smaller in size (arrowhead), the ventral commissure is missing, and the ventral white matter is disorganized or absent (black arrow). ***J–L***, Close up and quantification of central canal phenotype. Dorsal shift (*p *≤* *0.001, *d *=* *3.88; ***K***) and smaller size (*p *≤* *0.001, *d *=* *6.10; ***L***) of central canal in *hop* mice (*n* = 12) compared with controls (*n* = 10). ***M***, ***N***, Immunohistochemistry staining with NeuN in red and MBP in green confirms misplaced neurons in the gray matter of ventral spinal cord in *hop* mice at adult age (***N***) compared with controls (***M***). ***O***, Quantification of number NeuN**-**positive cells/section (*n* = 10 per genotype) in the lumbar white matter in the *hop* adult mice compared with control (*p *≤* *0.001, *d *=* *7.21). For brain morphology, refer to Extended Data Figure 2-1. The dotted line in ***E*** indicates the midline, and in ***M***, ***N***, the border between white and gray matter. Scale bars: 300 μm (***A***), 150 μm (***I***), 100 μm (***J***), and 50 μm (***C–E***, ***M***, ***N***). Significant effect of genotype **p <* *0.01, ***p ≤* *0.001.

Our electrophysiological experiments suggested that the aberrant locomotor phenotype arose from local spinal neuronal circuitry output, therefore we examined the morphology of the spinal cord. The gross morphology of wild-type postnatal spinal cords is characterized by a cervical and lumbar enlargement, which contain, among others, the neurons that coordinate forelimbs and hindlimb movement, respectively. The cervical enlargement extends from C5 to T1, and the lumbar enlargement extends from L_2_ to L_6_. The spinal cord from early postnatal control mice was found to measure 14 ± 3 mm. In *hop* mice, the length of the spinal cord, 14 ± 5 mm, and the size of the cervical enlargement were comparable to controls, whereas the lumbar enlargement was hardly detectable (Fig. 2*A*,*B*). Further, wild-type spinal cords had a visible ventral spinal artery fed by a radicular artery termed the artery of Adamkiewicz, which in wild-type mice usually originates at the level of L_1–2_ (Fig. 2*A*, arrow). In the lumbar region of *hop* spinal cords, the blood supply was abnormal, including the absence of a ventral spinal artery together with an undefined artery of Adamkiewicz (Fig. 2*A*, star).

10.1523/ENEURO.0518-21.2022.f2-1 Extended Data Figure 2-1 Brain commissure morphology appears normal in *hop* mice. Photomicrographs of horizontal sections from adult control (***A***, ***C***, ***E***, ***G***, ***I***, ***K***) and *hop* (***B***, ***D***, ***F***, ***H***, ***J***, ***L***) tissue. ***A***, ***B***, The corpus callosum (CC) and hippocampal commissure (HC) showed no gross defects. ***C***, ***D***, ***G***, ***H***, The anterior commissure (AC) also appeared normal. ***E***, ***F***, Sections showing the habenular commissure (HaC), the posterior commissure (PC), and the subcommissural organ (sc), all three present and with normal appearance in both control and *hop* mice. ***I***, ***J***, The pontine nuclei (pn) is also present in both genotypes. ***K***, ***L***, No aberrant commissure was found between the roof of the fourth ventricle (FV), and cerebellum (ce) at the junction of midbrain and hindbrain, in control and *hop* mice. Scale bars: 1000 μm (***A–D***, ***G***, ***H***) and 500 μm (***E***, ***F***, ***I–L***) Download Figure 2-1, TIF file.

Next, we used the spinal cord open-book preparation, in which the spinal cord is cut dorsally along the rostro-caudal axis, and the left and right halves of the spinal cord are dissected to reveal the inside of the two halves and the remaining ventral part. In this preparation, using NeuN as a marker for neurons, we found that the morphology of the lumbar part in *hop* mice was severely affected showing a diffuse border between the NeuN-positive neurons and the ventral area, whereas the more rostral and caudal parts were unaffected (Fig. 2*C*,*D*). Transverse sections of lumbar spinal cords stained with NeuN (gray matter) and MBP as a marker for myelinated axons (white matter), revealed less myelinated structures and misplaced neurons in *hop* mutants (Fig. 2*E*,*F*). Distribution plots obtained from these stainings further illustrate the misplacement of neurons along the medio-lateral axis (magenta) and a dorsally shifted central canal (blue) in *hop* mice (Fig. 2*G*,*H*).

Similarly, adult mice transverse sections of the cervical spinal cord from *hop* mice showed no morphologic differences compared with controls (Fig. 2*I*). However, sections of lumbar spinal cords from *hop* mice showed an altered morphology in the ventral spinal cord, including an undefined border between white and gray matter, the absence of a ventral funiculus and a dorsally shifted, poorly delineated, and smaller central canal (Fig. 2*I–L*). Immunohistochemistry, using NeuN and MBP antibodies, demonstrated misplaced neurons within the white matter also in adult *hop* mutants (Fig. 2*M–O*). Other mouse mutants with locomotor phenotypes have been reported to display severe defects in several brain regions, including the corpus callosum; hippocampal, anterior, habenular, and posterior commissure; as well as the pontine nucleus and cerebellar structures (Chiang et al., 1996; Serafini et al., 1996; Ackerman et al., 1997; Fazeli et al., 1997; Kullander et al., 2001; Páez et al., 2007). In contrast, morphologic analysis of brains from adult *hop* mice, which display enlarged ventricles likely connected to the hydrocephalic phenotype, did not show any further brain abnormalities (Extended Data Fig. 2-1).

Together, these findings suggest that the synchronous gait in *hop* mice is the result of ventral spinal cord alterations limited to the lumbar region responsible for controlling the hindlimbs. The rostro-caudal specificity is further supported by the observed forelimb alternating locomotion and normal cervical spinal cord morphology.

### Aberrant commissural fibers and neurotransmitter phenotype in *hop* mice

**Figure 3.**
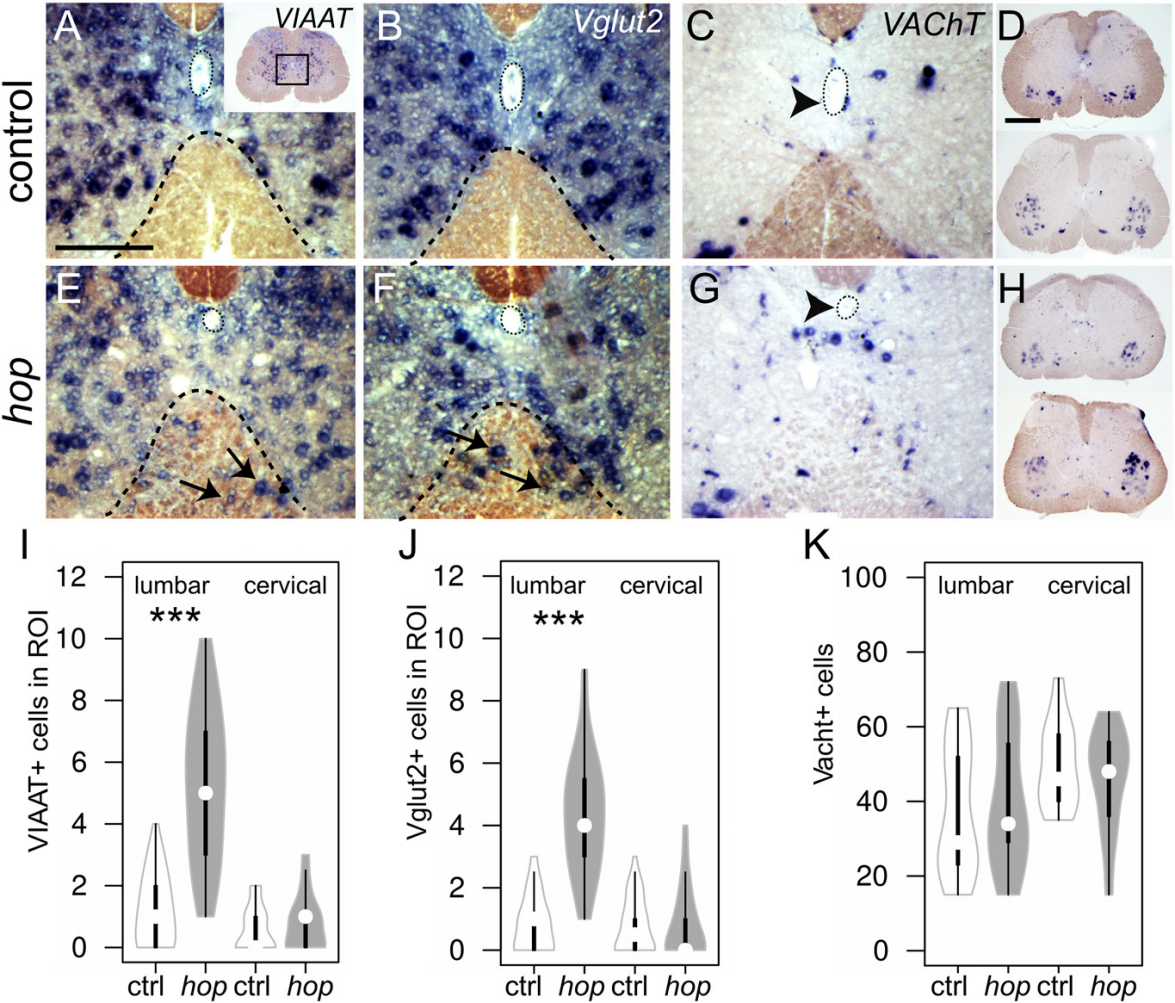
Neurotransmitter phenotype in the ventral spinal cord of postnatal *hop* mice. ***A–H***, *In situ* hybridization on adult 60-μm floating sections using probes against *Viaat* (***A***, ***E***), *Vglut2* (***B***, ***F***), and *VAChT* (***C***, ***D***, ***G***, ***H***). In control mice, neurons marked with either *Viaat* (***A***) or *Vglut2* (***B***) respect the border between white and gray matter, whereas in *hop* mice, *Viaat* (***E***) or *Vglut2* (***F***) mRNA**-**expressing neurons intermingle with the white matter. ROI as indicated with dotted black line. Number of displaced *Viaat*+ cells (*p *<* *0.001, *d* = −2.31; ***I***) and *Vglut2*+ (*p *<* *0.001, *d* = −3.16; ***J***) neurons in ROI are significant higher in lumbar but not cervical spinal cord (*p *≥* *0.05). *VAChT* stained cholinergic cells are found around the central canal in control (***C***) but are ventrally displaced relative to the central canal in *hop* mice (***G***). ***D***, ***H***, ***K***, Motor neuron position (LMC-MMC) and number are similar (***K***) in *hop* mice (***D***) and controls (***H***). *n* = 11–13 sections (L_1_ to L_3_) from *n* = 3 mice per genotype; Black box in ***A*** indicates area of enlargements. Neurons in the white matter are marked with black arrows. Arrowheads indicate central canal. Scale bars: 50 μm (***A–G***) and 150 μm (***D–H***). Significant effect of genotype ***p ≤* *0.001.

Cross-inhibitory and excitatory actions from CINs between the two sides of the locomotor CPG ensure coordinated left-right movements (Cowley and Schmidt, 1995; Kjaerulff and Kiehn, 1996). In an effort to further explain the aberrant locomotor phenotype, we examined the neurotransmitter profile of the neurons interspersed in the lumbar ventral white matter of *hop* mice using *in situ* hybridization. GABA/glycinergic, glutamatergic and cholinergic neurons were identified by detecting mRNA encoding the VIAAT, the vesicular glutamate transport (Vglut2), or the VAChT. We found both VIAAT and Vglut2-positive neurons in the ventral funiculus of *hop* mice demonstrating that misplaced neurons can be either excitatory or inhibitory (Fig. 3*A*,*B*,*E*,*F*,*I*,*J*). In addition, we found that central canal cholinergic cells were ventrally and medially displaced in *hop* mice compared with controls (Fig. 3*C*,*G*), whereas no apparent differences were found regarding position and number of motor neurons (Fig. 3*D*,*H*,*K*).

**Figure 4.**
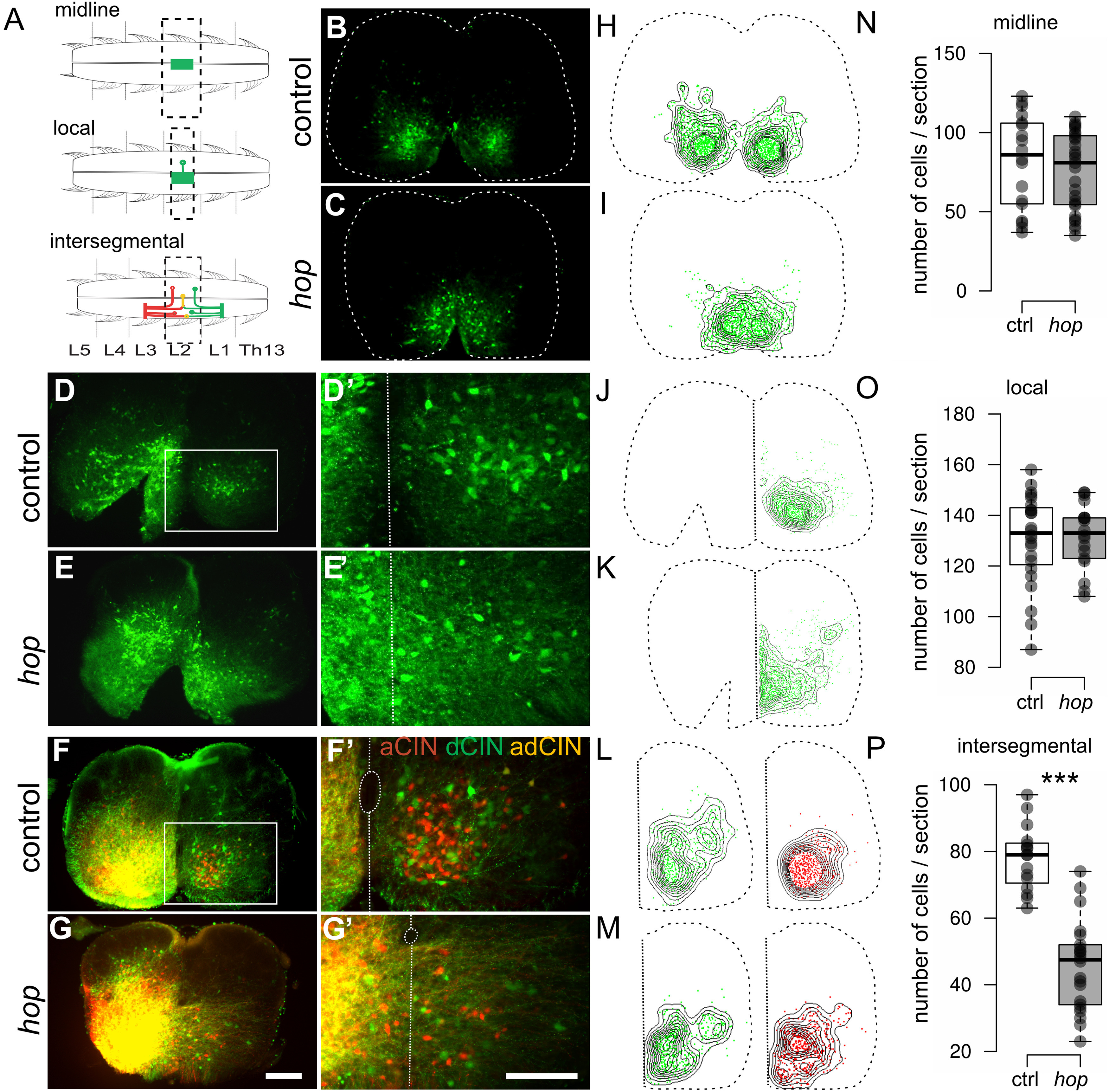
Aberrant commissural fibers and interneuron positioning in the ventral lumbar spinal cord of early postnatal (P0–P1) *hop* mice. ***A***, Schematics of different strategies for tracer application to detect CINs. Midline tracing using FDA to show local crossing CINs (***B***, ***C***), local tracing using FDA to label short projecting CINs (***D*–*E'***) or intersegmental FDA and RDA tracing to show ascending (aCINs), descending (dCINs), and bifurcating CINs (adCINs) in (***F*–*G'***). ***B***, ***C***, Transverse sections within the L_2_ level of spinal cords and density maps (***H***, ***I***) show interneuron clustering on the midline in *hop* mice (***C***, ***I***) as opposed to two ventromedial clusters in controls (***B***, ***H***). ***N***, Quantification of traced cells show similar number of cells found (*p *≥* *0.05; *n* = 9–12 sections from 3 mice per genotype). ***D–E'***, Transverse sections within the L_2_ level traced on L_2_ (***D***, ***E***) shows that the fibers freely cross the midline in *hop* (***E'***) compared with control mice (***D'***). ***J***, ***K***, Density maps illustrate diffuse and aberrant positioning of CINs on the midline in *hop* mice compared with controls. ***O***, Quantification of traced cells show similar CIN numbers (*p *≥* *0.05; *n* = 6–9 sections from 3 mice per genotype). ***F***, ***G'***, Aberrant fibers cross over the midline in spinal cords of *hop* mice (***G***, ***G'***) ventral from the central canal and the amount of long-range projecting CINs are reduced compared with controls. ***L***, ***M***, Density plots of aCINs (red) and dCINs (green) in control (***L***) and *hop* (***M***) spinal cords. ***P***, Quantification of traced cells show a significant reduction of intersegmental projections over the midline (RDA: *p *≤* *0.001, *d *=* *2.92, FDA: *p *≤* *0.001, *d *=* *2.96; aCIN, dCIN, and adCIN: *p *≤* *0.001, *d *=* *4.46; *n* = 8–11 sections from 4 mice per genotype). For intersegmental tracing on cervical spinal refer to Extended Data Figure 4-1. White box in ***D***, ***F*** indicates the area of close-up (***D'***, ***G'***). The outline of the spinal cord is indicated by a dashed line (***B***, ***C***), central canal and midline are indicated by a dotted line (***F'***, ***G'***). Scale bars: 100 μm (***B–G***) and 200 μm (***D'–G'***). Significant effect of genotype ***p ≤* *0.001.

Ventromedial CINs with axons crossing in the ventral commissure are necessary and sufficient for left-right coordination (Kjaerulff and Kiehn, 1996), which prompted us to investigate the CINs in *hop* mice. Short-range and long-range projecting CINs were examined by tracer applications of FDA and RDA in early postnatal mice (Fig. 4*A*). Midline retrograde tracing, using FDA to identify locally CINs, revealed that CINs at the lumbar level 2 (L_2_; Fig. 4*C*,*I*) cluster together on the ventral midline of *hop* mice compared with the two separate clusters of CINs seen in control mice (Fig. 4*B*,*H*), whereas similar numbers of local CINs were found (Fig. 4*N*). FDA application on one side of the L_2_ spinal cord illustrate a similar clustering of short projecting CINs on the ventral midline as well as aberrant fiber crossing (Fig. 4*D*,*E*). Despite altered positioning (Fig. 4*J*,*K*), the number of short-range projecting CINs in *hop* were similar compared with controls (Fig. 4*O*). To study the long-range projecting CINs, we examined three different subpopulations; the intersegmental ascending (RDA), descending (FDA), and bifurcating (RDA/FDA) CINs (Fig. 4*F–P*). In the cervical spinal cord (Extended Data Fig. 4-1), and in control mice also at the lumbar level, our tracings experiments showed that long-range projecting CIN populations clustered in the ventromedial area. At the lumbar level in *hop* mice, however, CINs were diffusely dispersed and partly located on the midline (Fig. 4*L*,*M*). In addition, lumbar long-range projection CINs were found to be reduced in *hop* compared with control mice (Fig. 4*P*).

10.1523/ENEURO.0518-21.2022.f4-1 Extended Data Figure 4-1 Normal CINs and axon midline crossing in the cervical spinal cord of early postnatal (P0–P1) *hop* mice. ***A–F***, Intersegmental tracing using FDA/RDA to locate ascending (aCINs), descending (dCINs), and bifurcating CINs (adCINs). No apparent defects in *hop* mice (***D–F'***) compared to controls (***A–C'***) on cervical sections were found. Download Figure 4-1, TIF file.

Taken together, these results show that, in *hop* mice, the ventromedial inhibitory and excitatory CINs responsible for normal left-right coordination cluster together on the ventral midline and that intersegmental CINs are partly lost and misplaced.

### An early developmental defect underlies the altered functionality in *hop* mice

**Figure 5.**
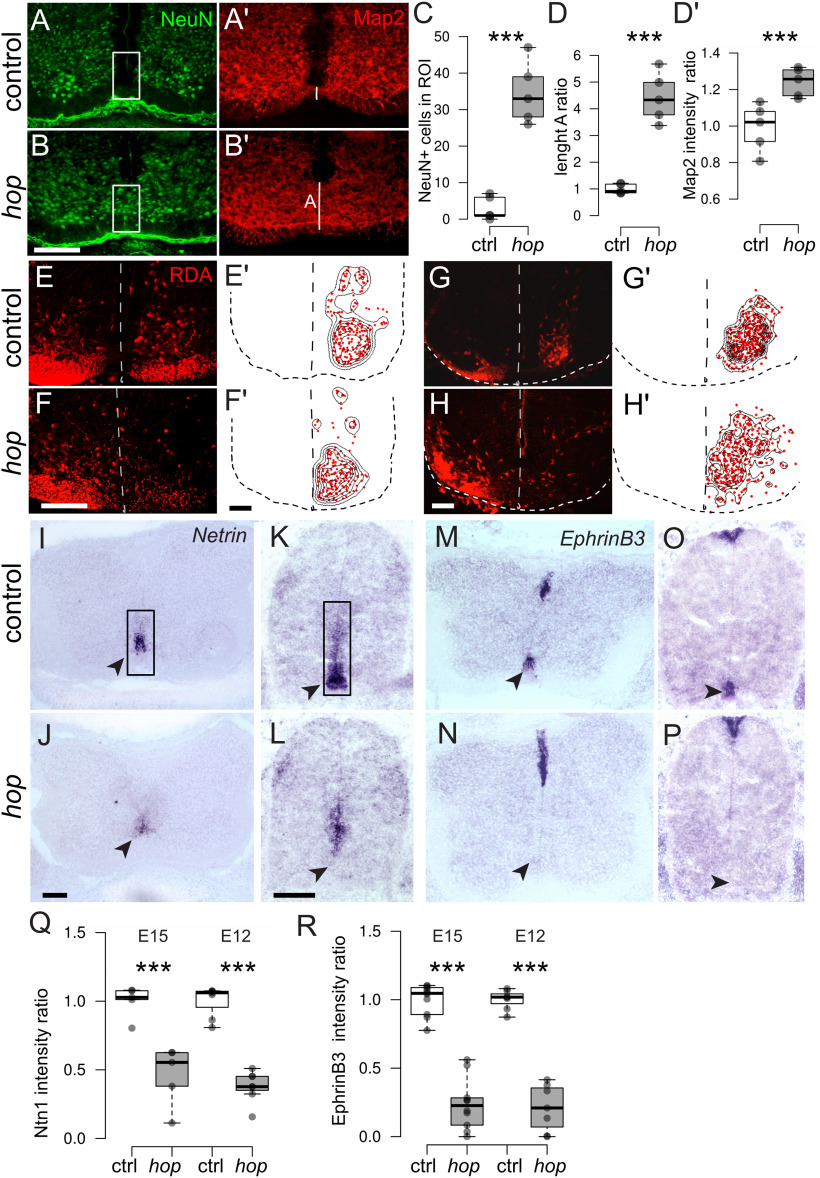
The distinct spinal cord phenotype in *hop* mice is present at embryonic stages. ***A***, ***B***, Photomicrographs of immunohistochemistry staining on E15.5 *hop* and control spinal cords with NeuN (green) and Map2 (red) showing aberrant fibers and misplaced neurons disrespecting the ventral midline. ***C***, ***D'***, White box in ***A***, ***B*** indicates ROI for statistical analysis of aberrant positioned NeuN+ cells and fiber crossing (***C***, NeuN+ cells: *p *<* *0.001, *d* = −5.46; ***D'***, Map2 intensity as ratio to control: *p *<* *0.001, *d* = −2.81; *n* = 5 per genotype). White line in ***A'***, ***B'*** indicates the thickness of the ventral commissure, quantified length as ratio to controls in ***D*** (*p *<* *0.001, *d* = −5.71; *n* = 5 per genotype). ***E–H***, RDA retrograde commisural interneuron (CIN) tracing on E15.5 (***E***, ***F***) and E12.5 (***G***, ***H***) spinal cord. Dotted line indicates midline and outline of the spinal cord. ***E'–H'***, Density plots visualizing medial displacement of traced CIN in *hop* (*n* = 12 sections from 3 embryos per genotype). ***I–P***, *In situ* hybridization on E15.5 (***I***, ***J***, ***M***, ***N***) and E12.5 (***K***, ***L***, ***O***, ***P***) lumbar spinal cords using probes detecting *Netrin1* and *EphrinB3*. Arrow heads point to regions of ventral *Netrin1* and *EphrinB3* mRNA expression. ***Q***, ***R***, Staining intensity (background corrected ratio) is significantly reduced in *hop* compared with control spinal cords for both axon guidance molecules and time points (*Netrin*; E12.5 *p *<* *0.001, *d *=* *5.86, *n* = 7 per group, E15.5: *p *<* *0.001, *d *=* *3.47, *n* = 5 per group; *EphrinB3*; E12.5 *p *<* *0.001, *d *=* *6.46, *n* = 7 per group, E15.5: *p *<* *0.001, *d *=* *5.19, *n* = 10 per group). Scale bars: 50 μm (***A–H***) 100 μm (***I–P***). Significant effect of genotype ***p ≤* *0.001.

CPG activity in rodents develops between E12 and birth. Correct assembly and maturation results in an established left-right coordination circuitry around birth (Branchereau et al., 2000; Nishimaru and Kudo, 2000; Nakayama et al., 2002). The prominent ventral spinal cord phenotype observed in postnatal *hop* mice prompted us to investigate the onset of the morphologic phenotype. Immunohistochemistry on E15.5 *hop* spinal cord tissue using microtubule associated protein 2 (Map2), to visualize fibers, and NeuN, to visualize neurons, revealed a similar fused spinal cord phenotype as seen in adult mice (Fig. 5*A–D*). In addition, using RDA tracing at E15.5 and E12.5, we found that the traceable CIN population in *hop* embryos was misplaced toward the midline and at E15.5 was more dispersed throughout the ventromedial spinal cord (Fig. 5*E–H*). These data indicate deviant ventral spinal cord formation presumably because of aberrant developmental processes, such as midline axon guidance.

Several synchronous locomotor phenotypes in mice have been associated with mutations involving midline axon guidance molecules, including Netrin1 and ephrinB3. The absence of the midline chemo-attractant Netrin1 leads to a complete switch from alternating to synchronous fictive locomotor activity (Rabe et al., 2009). Further, mutations in either the ligand ephrinB3 or the receptor EphA4 cause abnormal midline crossing of corticospinal axons and interneuron axons in the CPG, resulting in mice with a characteristic hopping gait phenotype (Kullander et al., 2003). To assess the expression of axon guidance molecules in the midline of *hop* mice, we performed *in situ* hybridization experiments on E15.5 and E12.5 spinal cord tissue with *Netrin1* and *EphrinB3* mRNA probes. The results showed that *Netrin1* expression levels were severely reduced in the lumbar ventral spinal cord of *hop* mice, with a clear loss in the floorplate itself, whereas remaining expression could be observed in the ventricular area. *EphrinB3* expression levels were also severely reduced in the ventral, but not dorsal spinal cord (Fig. 5*I–R*). These data suggest an early axon guidance defect in the ventral part of the spinal cord, which could at least partly explain the physiological phenotype in *hop* mice. However, guidance of commissural axons is not entirely dependent of floor-plate-derived Netrin1 (Moreno-Bravo et al., 2019), so the loss of Netrin1 in *hop* mice may relate more to earlier morphogenic events.

### Abnormal Shh and lumbar spinal cord patterning in *hop* mice

**Figure 6.**
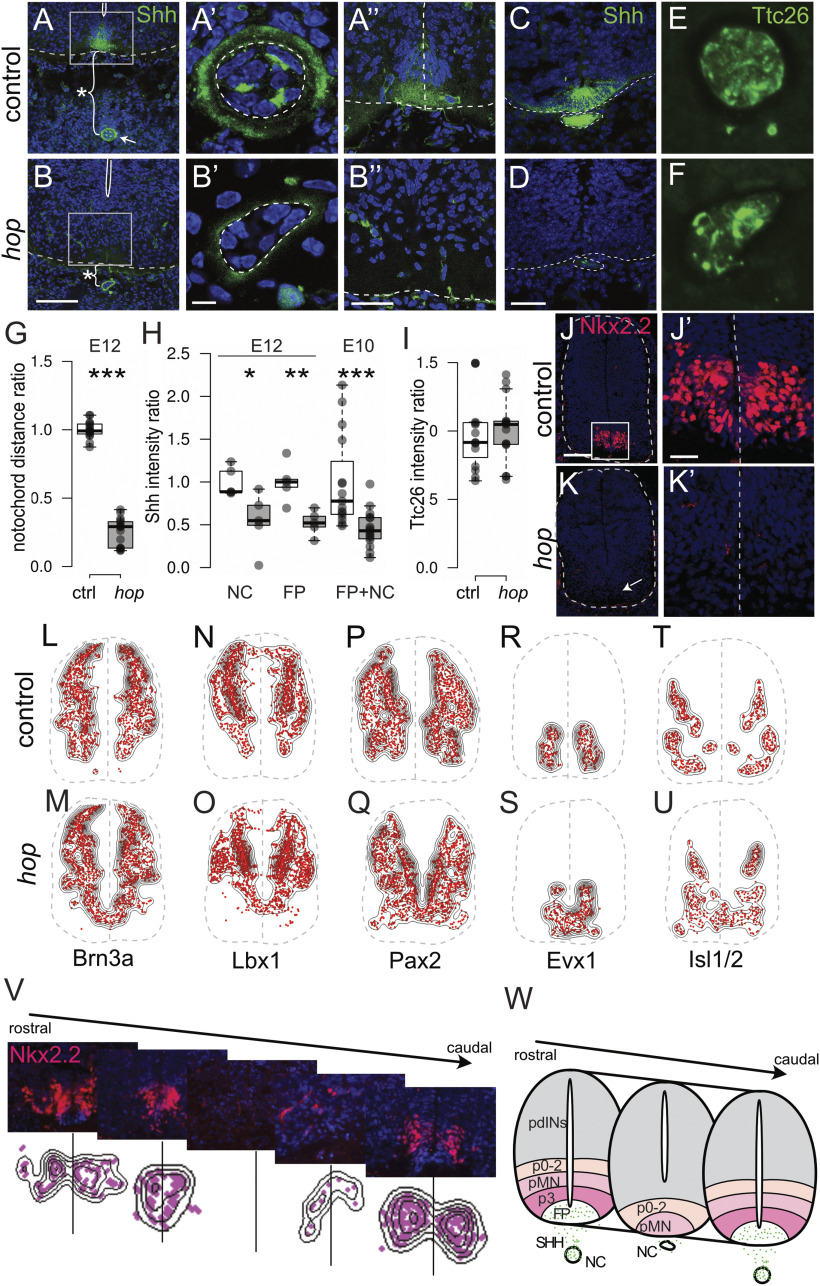
Abnormal Shh and lumbar spinal cord patterning in *hop* mice. ***A–D***, Photomicrographs of Shh immunohistochemistry staining on transverse spinal cord sections at E12.5 (***A***, ***B***) and E10.5 (***C***, ***D***). Notochord and floorplate phenotype at E12.5 in low power (***A***, ***B***) and close up from control (***A'***, ***A''***) and *hop* (***B'***, ***B''***) spinal cords. Dashed lines outline the spinal cord and notochord. The distance between the notochord and spinal cord is indicated by stars (***A***, ***B***) and is reduced in *hop* spinal cords (***G***; *p *<* *0.001, *d *=* *7.23, *n* = 15 per group). ***A'***, ***B'***, A malformed notochord together with reduced amounts of Shh (***H***; *p *=* *0.002, *d *=* *2.50, *n* = 5 per group). Lack of floorplate cells and severely reduced Shh expression in the spinal cord (***A''***, ***B''***) of *hop* mice compared with controls (***H***; *p *=* *0.016, *d *=* *1.93, *n* = 5 per group). ***C***, ***D***, Similarly, we found reduced amounts of Shh at E10.5 in *hop* spinal cords compared with controls (***H***; *p *<* *0.001, *d *=* *1.36, *n* = 15 per group). ***E***, ***F***, Ttc26 immunostaining on E12.5 spinal cord transverse sections. For clarity, blue nuclear counterstain with DAPI has been omitted. ***I***, The expression of Ttc26 is found in the notochord at similar intensity levels both in control and hop spinal cords. ***J–V***, Immunostainings on E12.5 spinal cord sections reveal a missing Nkx2.2 progenitor domain and a migration defect of neurons in the lumbar spinal cord of *hop* mice. ***J***, ***K***, The expression of the ventral most V3 progenitor marker Nkx2.2 is absent in lumbar sections from hop mice (***K***). White box indicates area of higher magnification (***J'***, ***K'***). ***V***, Nkx2.2 phenotype at different rostro-caudal levels exemplified with photomicrographs and visualized using density plots. The midline is indicated by lines (*n* = 4 sections per level). ***L–V***, Density plots show misplaced cells and lack of a ventral midline for additional progenitor markers as specified below each panel (*n* = 3). For representative photomicrographs, refer to Extended Data Figure 6-1. ***W***, Schematic summary of defects found in the *hop* mice in the lumbar part of the spinal cord including weak Shh expression, misplaced and malformed notochord, a dorsally shifted central canal, absence of floorplate and p3 domains as well as absence of a ventral midline. pdINs, progenitor domains for dorsal interneurons; FP, floor plate. The outline of the spinal cord and midline are indicated by dashed lines. FP, floorplate; NC, notochord. Scale bars: 100 μm (***A***, ***B***, ***J***, ***K***, ***M–V***), 25 μm (***A'***, ***B'***) 30 μm (***A''***, ***B''***, ***J', K'***), and 50 μm (***C***, ***D***). Significant effect of genotype *p <* *0.05, **p <* *0.01, ***p ≤* *0.001.

Next, we explored the expression of Shh, a key regulator of early development. Immunohistochemistry on E12.5 tissue with a Shh antibody revealed that the distance between the notochord and ventral spinal cord border was clearly decreased (Fig. 6*A*,*B*,*G*), and that Shh levels were reduced in both the notochord and spinal cord of *hop* mice (Fig. 6*H*). Strikingly, in the ventral spinal cord of *hop* mice, Shh expression was almost absent, likely as a result of a missing floorplate (Fig. 6*A’’*,*B’’*). Moreover, notochord morphology was abnormal displaying disorganized cells and low Shh expression (Fig. 6*A’*,*B’’*). Furthermore, Shh levels were also reduced in hop mutant embryos at E10.5 (Fig. 6*C*,*D*).

10.1523/ENEURO.0518-21.2022.f6-1 Extended Data Figure 6-1 Embryonic patterning defects in *hop* spinal cords. Photomicrographs of immunohistochemistry experiments on E12.5 spinal cord transverse sections using antibodies against transcription factors as indicated at the top. White boxes in ***A–E*** indicate areas of higher magnification in the respective panel below (***A'–E'***). The stainings show misplaced cells and lack of ventral midline for all tested progenitor markers. The outline of the spinal cord, central canal, and midline are indicated by dashed lines. Scale bars: 100 μm (***A–J***), 50 μm (***A'***, ***F'***), and 30 μm (***B'***, ***G'–J'***). Download Figure 6-1, TIF file.

**Figure 7.**
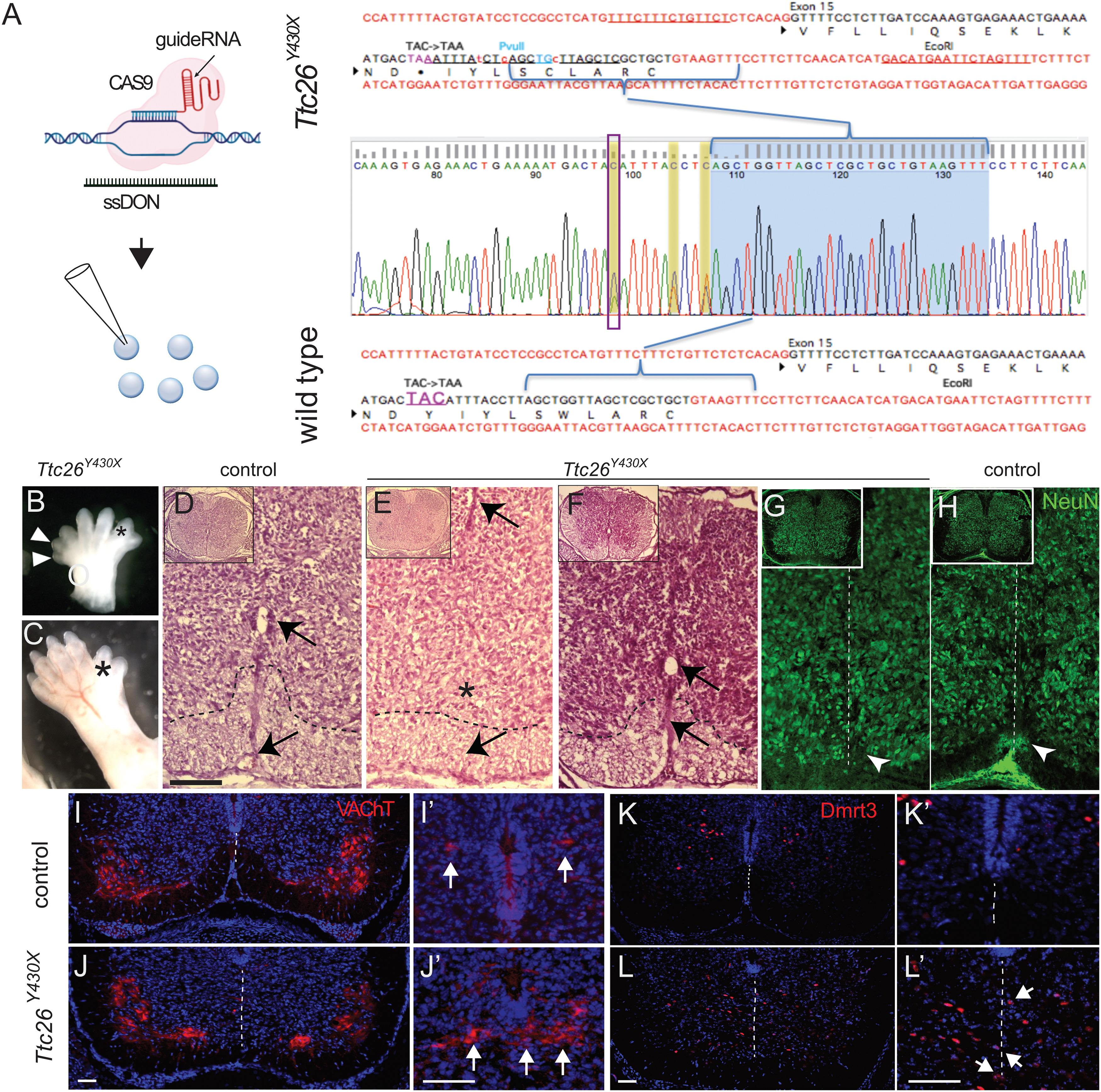
Introduction of a point mutation in *Ttc26* and analysis *Ttc26^Y430X^* mutants. For initial validation of the *hop* mouse mutation, refer to Extended Data Figure 7-1. ***A***, We then applied the CRISPR/Cas9 system to generate mice with a point mutation in exon 15 of the *Ttc26* gene, generating a substitution of tyrosine to a premature stop codon. By simultaneous microinjection of guideRNA, hCas9 mRNA and a single-stranded donor oligonucleotide (ssDON) into mouse oocytes, mouse chromosome six harboring the *Ttc26* gene was modified (left). The exon (black) and intron (red) organization is indicated together with Sanger sequencing traces of wild-type (bottom) and the mutated allele (top) highlighting the premature stop mutation. The introduction of a PvuII site for screening purposes (blue) and the location of the guideRNA are indicated. ***B***, ***C***, Images of E12.5 (***B***) and E16.5 (***C***) *Ttc26^Y430X^* mice showing preaxial polydactyly (arrowheads, star). ***D–F***, Photomicrographs of Hematoxylin and Eosin staining on E16.5 transverse spinal cord sections. ***D***, Lumbar spinal cord of control mice showing a well delineated central canal (upper black arrow), a present ventral funiculus with a border between white and gray matter (dashed line) and a present ventral spinal artery (lower arrow). ***E***, Lumbar spinal cord of *Ttc26^Y430X^* mice showing a poorly delineated dorsally shifted central canal (upper arrow), an absent ventral funiculus (upper star) and an absent ventral spinal artery (lower arrow). ***F***, Cervical spinal cord of *Ttc26^Y430X^* mice showing a well delineated central canal (upper arrow) and a present ventral funiculus with a clear midline border (lower arrow). ***G–L***, Photomicrographs of immunohistochemistry experiments on E16.5 transverse lumbar spinal cord sections of control and *Ttc26^Y430X^* mice. ***G***, NeuN staining showing a poorly defined ventral midline in *Ttc26^Y430X^* mice, with misplaced neurons within the white matter of the ventral funiculus. Enlargements on the right show cells positioned at the location of the missing midline in mutant but not control spinal cord sections (***G***, ***H***). ***I***, ***J***, Cholinergic neurons as identified by their VAChT expression (red) were found in the medial and lateral motor neuron cluster, as well as in neurons around the central canal. Dmrt3**-**expressing interneurons were found medioventrally from the central canal. In mutant *Ttc26^Y430^* spinal cords, medial and lateral motor neuron nuclei were located at their expected positions. In contrast, central canal cholinergic neurons and Dmrt3**-**expressing interneurons were found ventral of the central canal and positioned in close proximity or even on top of the presumed position of the midline. ***I***, ***J***, VAChT immunostaining showing no apparent differences regarding position and size of motor neurons between *Ttc26^Y430^* and control mice. ***I'***, ***J'***, Central canal VAChT-expressing cells, as well as (***K***, ***L***) Dmrt3**-**expressing cells, were ventrally and medially displaced in *Ttc26^Y430^* spinal cords compared with controls. ***J'***, ***L'***, Enlargement of the ventromedial area of the spinal cord demonstrating misplaced cells in *Ttc26^Y430^* spinal cords (***J'***, arrows). Scale bars: 100 μm (***D–H***), 50 μm (***I–L***), **and** 100 μm (***I'–L'***). For comparative analysis in the rabbit, refer to Extended Data Figure 7-2.

10.1523/ENEURO.0518-21.2022.f7-1 Extended Data Figure 7-1. Validation of the *hop* mouse mutation. The exon/intron organization of *Ttc26* is indicated together with Sanger sequencing traces of wild type and *hop* highlighting the premature stop mutation in exon 15 (out of 18) of *Ttc26*. Genomic DNA from a *hop* homozygote (stock 002718) and BALB/cByJ control (stock 001026) was obtained from The Jackson Laboratory’s DNA Resource (*n* = 3 per genotype). Exomes were captured at the Yale Center for Genome Analysis, using the NimbleGen SeqCap EZ Mouse Exome and following NimbleGen protocols. Captured pools were sequenced (75 bp, paired-end) on an Illumina HiSeq 2000 using previously described methods (Choi et al., 2009). We obtained ∼91 million (BALB/cByJ) to ∼113 million (*hop*) high-quality reads. Illumina reads were first trimmed based on their quality scores to remove low-quality regions using the program Btrim (Kong, 2011). A cutoff of 20 for average quality scores within a moving window of size 5-bp was used. Minimum acceptable read length was 25 bp. Other parameters of Btrim were set to defaults. The preprocessed reads were then aligned to the mouse genome reference sequence (mm9) using the BWA mapping program (Li and Durbin, 2009). The mapping results were converted into SAMtools pileup format using SAMtools programs (Li et al., 2009). PCR duplicates were removed using the rmdup command from SAMtools, resulting in ∼84x (BALB/cByJ) or ∼100x (*hop*) coverage across the exome. More than 97% of all bases included in the exome showed at least 8x coverage and >90% of the bases showed at least 20x coverage. Single nucleotide variations (SNVs) were called using SAMtools’ pileup command. Further filtering was performed using in-house scripts to exclude those SNV calls that had less than 3 reads or a SNP score less than 20. Annotation was added based on the UCSC RefSeq gene model (http://genome.ucsc.edu/; Pruitt et al., 2009). Based on SNV homozygosity mapping, the interval was narrowed to ∼700 kb on chromosome 6 (37.9–38.6 Mb), which fell within the previously mapped *hop* genomic region. After filtering for known SNPs and repeat regions, we identified 17 possible point mutations, 16 of which were located in introns or 3′ untranslated regions. Download Figure 7-1, TIF file.

A point mutation in the *Ttc26* gene was found in a previous study of *hop* mice (Swiderski et al., 2014). *Ttc26* has been classified as an intraflagellar transport (IFT) complex B protein (Follit et al., 2009). Studies conducted on other IFT mutants have revealed phenotypic defects similar to those in the *hop* mutant including ventral spinal cord patterning defects, loss of ventral cell types and polydactyly (Huangfu et al., 2003; Liu et al., 2005; Haycraft et al., 2007). Therefore, we evaluated protein expression of TTC26 using immunohistochemical analysis and antibodies against TTC26 in the early developing spinal cord (Fig. 6*E*,*F*). Despite the apparent improper shape and positioning of the notochord, at E12.5, we found that the truncated TTC26 protein was still detectable in *hop* mice (Fig. 6*F*,*I*).

Loss of Shh in mice results in degeneration of the notochord, absence of a morphologically distinct floorplate and abnormal dorsoventral patterning (Chiang et al., 1996). Consequently, we analyzed embryonic spinal cords of *hop* mice using antibodies against transcription factors as markers for specific developmental subpopulations to assess patterning defects (Fig. 6*J–V*; Extended Data Fig. 6-1; Moran-Rivard et al., 2001; Gross et al., 2002). Most striking, this analysis revealed that the ventral most V3 progenitor domain labeled by Nkx2.2, although present in controls was undetectable in *hop* mice (Fig. 6*J*,*K*). The complete loss of Nkx2.2-positive cells in *hop* mice was restricted to the lumbar level and was found to be less apparent with increasing distance in both rostral and caudal directions and again, these defects were not found in the thoracic region (Fig. 6*V*). Further, we used Brn3a to examine the dorsal subpopulations dI1– dI3 and dI5 interneurons, which were largely unaffected, except in the most ventral regions (Fig. 6*L*,*M*; Extended Data Fig. 6-1*A,F*). Similarly, the dI4–dI6 subpopulations labeled by Lbx1 (Fig. 6*N*,*O*; Extended Data Fig. 6-1*B,G*) and the dI4, dI6, p0, and p4 subpopulations labeled by Pax2 (Fig. 6*P*,*Q*; Extended Data Fig. 6-1*C,H*) were present and appeared normal, except for the ventral-most labeled cells, which were found in close vicinity to the midline in *hop* mice. Markers for ventral V0v cells labeled by Evx1 and for dI3 and motor neurons labeled with Isl1/2 identified cells residing on and close to the ventral midline in *hop* mice (Fig. 6*S*,*U*; Extended Data Fig. 6-1*I,J*), which was not seen in controls (Fig. 6*R*,*T*; Extended Data Fig. 6-1*D,E*). Thus, our results show that there is an early patterning defect specific to the lumbar spinal cord of *hop* mice, likely originating from a defect in the notochord early organizer.

### Validation of the *hop* mutation

In the *hop* mouse, defects that are associated with early developmental patterning and Shh signaling are apparent, supporting the hypothesis that the identified point mutation in *Ttc26* is the causative mutation, and in line with a previous study (Swiderski et al., 2014). Our whole exome sequencing of a *hop* mouse and of *Ttc26* exon 15 in several heterozygous and homozygous individuals confirmed a C to A transversion at position 38412065 bp causing a premature stop codon and a truncated form of the TTC26 protein (Extended Data Fig. 7-1). However, TTC26 truncated protein remains expressed and it is possible that other mutations are present in neighboring genes, which may cause the observed phenotype. In the *hop* mutation area and in the close vicinity to the *Ttc26* gene, genes that have been associated with gait defects can be found. For example, *Hipk2* has been associated to TGF-β signaling, another influential spinal cord morphant that plays an essential role in the regulation of survival in the nervous system (Doxakis et al., 2004; Isono et al., 2006; Zhang et al., 2007). Ablation of *Hipk2* generated mice with an ataxic-like phenotype implying that the *Hipk2* gene may contribute to the *hop* phenotype (Anzilotti et al., 2015).

We sought to exclude such additional possible causative mutations and determine whether the underlying genetic cause in the *hop* mouse is solely because of the identified mutation in *Ttc26*. Using clustered regularly interspaced short palindromic repeat/CRISPR-associated 9 (CRISPR/Cas9) technology, we introduced a point mutation within *Ttc26* exon 15 at position 38412065 bp in wild-type mice (Fig. 7*A*). The generated CRISPR *Ttc26^Y430X^* mutant mice displayed polydactyly of all feet (Fig. 7*B*,*C*), highly reminiscent of *hop* mice. However, whereas previous reports found a partial lethality between E10.5 and birth for *hop* mutant mice, we failed to receive live born homozygous *Ttc26^Y430X^* pups (Bryan et al., 1977). Among 67 delivered pups from nine heterozygous breedings, we received one live-born homozygous pup that died early. We therefore performed our phenotypic analysis of embryonic stages.

10.1523/ENEURO.0518-21.2022.f7-2 Extended Data Figure 7-2 Relatively high number of missense mutations in the rabbit *Ttc26* gene. ***A***, Amino acid alignment of TTC26 sequences from eight mammals made using MUSCLE with missense mutations in rabbit marked (pink boxes). ***B***, Photomicrograph of HE stained lumbar spinal cord section from wild-type adult rabbit (box shown magnified region). The morphology is similar to the wild-type mouse spinal cord, with a well delineated central canal (upper arrow), a ventral funiculus with a clear border between white and grey matter and a ventral spinal artery (lower arrow). ***C–G***, Immunohistochemistry performed on postnatal rabbits towards NeuN (***C***), Chat (***D***), Lbx1 (***E***), Parvalbumin (***F***), and Pax2 (***G***) show a clear bilateral separation of the spinal neuronal populations. Scale bars: 200 μm (***C–G***). Download Figure 7-2, TIF file.

CRISPR *Ttc26^Y430X^* mutant embryos showed a spinal cord phenotype similar to the *hop* mice (Fig. 7*B–H*). Ventral spinal cord morphology matched the *hop* mice, with a poorly delineated and dorsally shifted central canal (Fig. 7*E*,*F*). Furthermore, like in *hop* mutants, defects were regionalized to the lumbar spinal cord with the cervical region unaffected (Fig. 7*D*). NeuN immunolabeling to identify neurons corroborated that the border between the white and gray matter was poorly defined, as was found in *hop* mutants (Fig. 7*G*,*H*). There were also less myelinated structures and misplaced neurons within the white matter, resulting in an absent ventral funiculus (Fig. 7*G*). These findings demonstrate that the spinal cord phenotype found in *hop* mice was mirrored in *Ttc26^Y430X^* mice, at least for embryonic stages. Thus, the C-to-A point mutation in the *Ttc26*-gene, exon 15 is likely the sole cause for the observed spinal cord defects in *hop* mice.

Finally, we sought to investigate how two populations of importance for locomotion were affected: motor neurons and a portion of the dI6 inhibitory interneurons defined by Dmrt3 expression (Andersson et al., 2012; Perry et al., 2019). Immunohistochemistry using primary antibodies against VAChT were used to label motor neurons in the lumbar part of *Ttc26^Y430X^* and control spinal cords at E16.5. Quantification gave a mean value of 19.6 ± 9.4 motor neurons per hemisection (51 hemisections counted) in the *Ttc26^Y430X^* spinal cord and 20.6 ± 9.3 motor neurons per hemisection (57 hemisections counted) in the control, which was not significantly different (independent-samples Student’s *t* test; *p* = 0.60). Examination of the motor neuron distribution in the lumbar region of the Ttc26^Y430X^ spinal cord revealed two distinct populations on each side of the midline, similar to controls (Fig. 7*I*,*J*). This indicated that, despite the midline fusion in the ventral spinal cord, the left and right populations of motor neurons remained separate, as was also found in the adult *hop* mutants (Fig. 3). In contrast, central canal cholinergic cells, that are normally separated by the midline, was distributed closer to and even on the midline in the Ttc26^Y430X^ spinal cord (Fig. 7*I'*,*J’*). We next explored the distribution of dI6 interneurons using anti-Dmrt3 antibodies. As expected, in controls they were located medioventrally in the spinal cord and formed two distinct populations on each side of the midline. However, in *Ttc26^Y430X^* spinal cords, the Dmrt3-positive cells were clustered on or close to the midline and positioned further ventrally (Fig. 7*K*,*L*,*K’*,*L’*).

## Discussion

Here, we analyzed *hop* mice, focusing on the underlying cause for their aberrant locomotor phenotype. Behavioral tests and fictive locomotion experiments showed that *hop* mice move using their hindlimbs in synchrony. Our developmental studies revealed an abnormal notochord, a missing floorplate and the absence of a Nkx2.2 progenitor domain in *hop* mice as well as a disorganized dorsoventral patterning with neurons residing on the ventral midline. Further, we found reduced expression of the axon guidance molecules netrin-1 and ephrinB3. By verifying the causative mutation in *Ttc26*, we revealed its important role for the formation of a lumbar locomotor network containing two separate CPG half-centers.

### Lack of ventral spinal cord midline explains the synchronous gait

According to the half-center model of the spinal locomotor CPG proposed by Graham-Brown (Graham-Brown, 1911), the rhythmic pattern of alternating bursts of flexor and extensor muscles is produced by neuronal populations in each half of the spinal cord. Commissural projections by inhibitory and excitatory neurons ensure normal left-right coordination (Kiehn, 2006). After cutting the ventral commissure, the normal left-right circuit disappears indicating that ventromedially located CINs are indispensable for the bilateral coordination (Kjaerulff and Kiehn, 1996; Restrepo et al., 2009). Our morphologic studies of the spinal cord in *hop* mice revealed defects in the ventral spinal cord, including an absent midline and a loosely defined white/gray matter border. The disturbed expression of axon guidance molecules in *hop* mice are likely to underlie the observed aberrant fibers of short-, long-, and locally-projecting INs. Furthermore, their cell bodies were spread on the ventral midline compared with the two separate ventromedial clusters of CINs seen in control mice (Fig. 4). Also, both excitatory and inhibitory neurons were found to reside on the ventral midline (Fig. 3). Midline defects were discernable as early as E12.5, where we found that ventral neuronal progenitors in the lumbar spinal cord were located where the midline should have been (Fig. 6). These results indicate that in *hop* mice, the neurons that normally form two separate CPG half-centers, instead intermingle. This would cause a functionally fused CPG center, explaining the observed synchronous gait.

Expression of prepattern genes along the dorsoventral axis of the spinal cord is controlled by long range signals from the opposite poles of the neural tube. *Sonic Hedgehog*, *Gli3*, and *Smoothened* are among the most important molecules for ventral cell differentiation, with the most ventral progenitor domain requiring the highest concentration of *Shh* to develop normally (Placzek, 1995; Briscoe and Ericson, 1999). Already at E10.5, the most ventral Nkx2.2 progenitor domain, which gives rise to V3 neurons, was absent in the *hop* mice. V3 neurons are one of the major classes of excitatory CINs in the mouse spinal cord and are components of the locomotor network, with a role in establishing a stable and balanced locomotor rhythm (Zhang et al., 2008; Danner et al., 2019).

Motor neuron populations, however, were not fused at the midline in *hop* mice. Previous studies have shown that the upper edge of the Islet1-expressing motor column is dorsally shifted in Netrin-1 mutant spinal cords at E10 (Kim et al., 2015). In *hop* mice, at E12.5, cells expressing Islet1 were dispersed dorsally and clustered together on the ventral midline (Extended Data Fig. 6-1). Likewise, Robo mutant embryos (E9.5–E10.5) have a pack of misplaced Islet1^+^ motor neurons within the floorplate. However, the ectopic Islet1^+^ cells of Robo mutants are no longer visible in the floorplate by E12.5. Our study of the *Ttc26^Y430X^* spinal cords at E16.5 suggest that decreased levels of Netrin-1 in fact do affect the initial positioning of motor neurons, but that compensatory mechanisms then correct the misplacing before birth. We did not find a significant difference between the number of motor neurons in *Ttc26^Y430X^* and control spinal cords, which indicates that the misplaced neurons most likely migrate back to their correct location, rather than undergo apoptosis. In addition to Semaphorin and Slit signaling governed by the transcription factors Islet1 and Islet2 (Mauti et al., 2007; Lee et al., 2015), motor neuron positioning is regulated by Reelin and Cadherin signaling causing a secondary reorganization of motor columns into sub-clusters consisting of motor pools (Price et al., 2002; Palmesino et al., 2010; Demireva et al., 2011). The migration and positioning of motor neurons seems to be more strictly regulated than the positioning of CINs, possibly explaining why motor neurons are not clustered on the ventral midline of *hop* and *Ttc26^Y430X^* spinal cords. Further studies are needed to understand the exact underlying mechanisms for the observed differences between motor neuron and interneuron development in the ventral spinal cord.

### Rostro-caudal specificity of the midline fusion

In addition to the previously reported characteristic hopping gait in *hop* mice (Johnson and Hunt, 1971; Wojnowski et al., 1998), our gait analysis showed that adult *hop* mice alternated their forelimbs while they moved their hind limbs in synchrony. Further, we found that the pattern of flexor-extensor alternation during fictive locomotion was maintained following hemisection, suggesting that the circuitry coordinating ipsilateral alternation is intact in *hop* mutants (Extended Data Fig. 1-1). However, we did observe a tendency for asymmetry in flexor and extensor alternation (Fig. 1*H*; Extended Data Fig. 1-1*A*), suggesting that a bias toward extension exists, possibly because of a reduction in inhibitory drive from the flexor-related module of the CPG (Kiehn et al., 2008). This may be because of a reduced number of inhibitory descending CINs, or alternatively, ipsilateral flexor inhibitory projections onto extensor motoneurons. Even if we found a reduced number of traceable intersegmental CINs, both these scenarios remain possible and are not mutually exclusive. These findings suggest that the defect behind the characteristic hopping phenotype is limited to the lumbar spinal cord controlling the left-right alternation of the hind limbs, and possibly descending INs that affect coordination. This is in accordance with the lumbar specific defects observed in *Ttc26* mutant mice early in development.

What might be the possible explanation for the rostro-caudal specificity of the defect? Primary cilia and Shh signaling are critical for proper patterning of the neural tube along the dorsoventral axis (Tarchini et al., 2006; Dessaud et al., 2008), and TTC26 is required for accumulation of Gli at the ciliary tip (Swiderski et al., 2014). In Shh KO mice, motor neurons are not generated, however, in Shh and Gli3 double KO mice there is a rescue effect where motor neurons are generated predominantly in the lumbar region (Litingtung and Chiang, 2000). This substantiates the involvement of the Shh signaling pathway in differential development along the rostral-caudal axis, possibly because of an unknown caudal factor such as retinoic acid (Litingtung and Chiang, 2000). Interestingly, missense mutations in the *ttc26* gene are more frequent in the rabbit compared with other mammals with alternating gaits (Extended Data Fig. 7-2*A*). To investigate whether rabbits might have a rearranged lumbar region in their spinal cord explaining their synchronous hindlimb gait, we analyzed rabbit tissue. There were, however, no signs of ventral fusion in the postnatal lumbar spinal cord (Extended Data Fig. 7-2*B*) and spinal neuron populations were clearly separated (Extended Data Fig. 7-2*C–G*), suggesting that mutations in *Ttc26* are not underlying the synchronous gait in rabbits.

### The mutation in *Ttc26* is causative for the spinal cord fusion

Shh is crucial for induction of both floorplate and dorsoventral patterning of the spinal cord (Placzek, 1995; Chiang et al., 1996; Patten and Placzek, 2000), and in particular, defective secretion of Shh can result in the spinal cord developing without floorplate cells and V3 interneurons (Iulianella et al., 2018). *Shh* mutant mice have several defects in midline structures such as the notochord and cyclopia (Boije et al., 2012). A similar spinal cord phenotype is present in HNF-3β null mutant mice. HNF-3β is a transcription factor expressed in the three midline organizing centers; the node, notochord and floorplate (Ang and Rossant, 1994).

Similar to Swiderski et al. (2014), our positional cloning analysis localized the hop mutation to *Ttc26*. TTC26, a component of IFT complex B, is a structure necessary for cilia formation and protein transport, and is important for Shh signaling. In addition, Ttc26 (also named IFT56) regulates vertebrate developmental patterning by maintaining IFTB complex integrity and ciliary microtubule architecture (Xin et al., 2017). Moreover, cilia motility is important for embryonic left-right determination (Nonaka et al., 1998). Although it is likely that the found mutation is the causative one, we noticed some differences between our analysis and the Swiderski et al. (2014) study. The spinal cord ventralization was found in both studies, but additionally, we found midline fusion defects as well as lack of the ventral midline and Nkx2.2-positive V3 progenitors in the lumbar spinal cord. These differences opened the possibility of other additional mutations in the *hop* mice. For example, *Gli1^−/−^*; *Gli2^+/−^* mice have a hopping gait (Park et al., 2000), and *Gli3^+/−^* mice have preaxial polydactyly (Hui and Joyner, 1993; Park et al., 2000). In addition, a similar distortion of notochord, midline fusion (E14) and missing Nkx2.2 domain has been observed in *Gli1/2* double mutants. Also, in *Gli2*−/− mouse embryos, the most ventral cells (the floor plate) are not specified (Park et al., 2000).

The lower expression levels of Shh seen in *hop* mice could also be caused by mutations in upstream regulators of Shh, events that could take place earlier in development. As mentioned above, the HOX1 cluster, located on cytoband B3-C >25.40 cM in the vicinity of the *hop* region, could be affected in the *hop* mouse mutation. Mice with homozygous mutations in one or multiple members of the cluster display a range of phenotypes including developmental defects in the skeletal, reproductive and nervous systems (Raines et al., 2013). Two other genes in the *hop* region are associated to gait defects or could explain a fused spinal cord midline. When the *Braf* gene is mutated it has been reported to cause developmental deficits such as craniofacial dysmorphism and extra digits (Moriya et al., 2015). Mice with a targeted mutation in the transcriptional cofactor homeodomain interacting protein kinase 2 (HIPK2) display abnormal gaits including shuffling, reduced width and short stride length (Zhang et al., 2007). For this reason, we introduced a point mutation in the *Ttc26* gene, which produced a premature stop codon mimicking the *hop* mouse mutation. Unfortunately, potentially because of different genetic backgrounds, unlike *hop* mutants, *Ttc26^Y430X^* mutants displayed fully penetrant embryonic lethality, why locomotor studies could not be performed. However, *Ttc26^Y430X^* mutant mice displayed the same spinal cord ventral midline fusion as was found in *hop* mice, suggesting that this point mutation can sufficiently explain the observed mutant phenotype.

In conclusion, our findings suggest that the aberrant spinal cord phenotype observed in *hop* mice is the result of abnormal developmental processes, including induction from the notochord and Shh signaling. Despite the loss of the ventral midline, functional neuronal networks develop and form connections able to produce coordinated, albeit synchronous, activity. Our data support the two-half-center hypothesis by demonstrating that midline separation is crucial for left-right alternation of locomotion. To our knowledge, the *hop* mutant is the first model with a specific ventral spinal cord fusion, in which the components of the CPG can be studied and understood.

## Synthesis

Reviewing Editor: Silvia Pagliardini, University of Alberta Decisions are customarily a result of the Reviewing Editor and the peer reviewers coming together and discussing their recommendations until a consensus is reached. When revisions are invited, a fact-based synthesis statement explaining their decision and outlining what is needed to prepare a revision will be listed below. The following reviewer(s) agreed to reveal their identity: Avihu Klar. Note: If this manuscript was transferred from JNeurosci and a decision was made to accept the manuscript without peer review, a brief statement to this effect will instead be what is listed below.

The authors of the current manuscript systematically characterized the morphological and some physiological phenotypes of the hop mice. They revealed that a fused lumbar spinal cord with massive cell patterning and axon guiding defects led the left-right synchronization of the locomotor output. Also interestingly, they confirmed that the point mutation in ttc26 gene was sufficient to cause the morphological defects. There are a couple of minor points for the authors to consider.

1. The morphological phenotypes in hop mice are very interesting. I agree with previous reviewer 2, however, with such massive abnormal cell patterning during development, this may not be a good model system to study regular CPG circuits. The current results do not prove or disprove the ‘half-center’ theory. However, they provide evidence that support this theory. They do demonstrate that several spinal interneurons populations collapse to the midline.

2. Figure 1. According to the time scale of their representative traces, the oscillation frequency of the mutant cords would be much slower than those in the wildtype, but their data didn’t show that. I wonder if they picked the wrong trace or the scale bars were wrong. Missing letters C and E in the Fig 1

3. lines 277-279: The reference indicated in line 278 describes the guidance of axons in the hindbrain. The references that describe the guidance of comm axons in the floor plate are:

-Wu, Z., Makihara, S., Yam, P.T., Teo, S., Renier, N., Balekoglu, N., Moreno-Bravo, J.A., Olsen, O., Chedotal, A., Charron, F., and Tessier-Lavigne, M. (2019). Long-Range Guidance of Spinal Commissural Axons by Netrin1 and Sonic Hedgehog from Midline Floor Plate Cells. Neuron 101, 635-647 e634.

-Moreno-Bravo, J.A., Roig Puiggros, S., Mehlen, P., and Chedotal, A. (2019). Synergistic Activity of Floor-Plate- and Ventricular-Zone-Derived Netrin-1 in Spinal Cord Commissural Axon Guidance. Neuron 101, 625-634 e623.

-Varadarajan, S.G., Kong, J.H., Phan, K.D., Kao, T.J., Panaitof, S.C., Cardin, J., Eltzschig, H., Kania, A., Novitch, B.G., and Butler, S.J. (2017). Netrin1 Produced by Neural Progenitors, Not Floor Plate Cells, Is Required for Axon Guidance in the Spinal Cord. Neuron 94, 790-799 e793.

4. In addition, the statement: “guidance of commissural axons does not require floor-plate-derived Netrin1", is partially correct. I find that the above Moreno-Bravo et al., paper describes best the relative contribution of the floor plate-derived Netrin.

5. Please check the spelling throughout the text to eliminate typos.

## Author Response

Response letter

The authors of the current manuscript systematically characterized the morphological and some physiological phenotypes of the hop mice. They revealed that a fused lumbar spinal cord with massive cell patterning and axon guiding defects led the left-right synchronization of the locomotor output. Also interestingly, they confirmed that the point mutation in ttc26 gene was sufficient to cause the morphological defects. There are a couple of minor points for the authors to consider.

1. The morphological phenotypes in hop mice are very interesting. I agree with previous reviewer 2, however, with such massive abnormal cell patterning during development, this may not be a good model system to study regular CPG circuits. The current results do not prove or disprove the ‘half-center’ theory. However, they provide evidence that support this theory. They do demonstrate that several spinal interneurons populations collapse to the midline.

Thanks for this comment. We agree that we provide evidence that support the “half-center” theory, which is what we state in the conclusion.

2. Figure 1. According to the time scale of their representative traces, the oscillation frequency of the mutant cords would be much slower than those in the wildtype, but their data didn’t show that. I wonder if they picked the wrong trace or the scale bars were wrong. Missing letters C and E in the Fig 1

Yes, quite right, we have updated figure 1 accordingly.

3. lines 277-279: The reference indicated in line 278 describes the guidance of axons in the hindbrain. The references that describe the guidance of comm axons in the floor plate are:

-Wu, Z., Makihara, S., Yam, P.T., Teo, S., Renier, N., Balekoglu, N., Moreno-Bravo, J.A., Olsen, O., Chedotal, A., Charron, F., and Tessier-Lavigne, M. (2019). Long-Range Guidance of Spinal Commissural Axons by Netrin1 and Sonic Hedgehog from Midline Floor Plate Cells. Neuron 101, 635-647 e634.

-Moreno-Bravo, J.A., Roig Puiggros, S., Mehlen, P., and Chedotal, A. (2019). Synergistic Activity of Floor-Plate- and Ventricular-Zone-Derived Netrin-1 in Spinal Cord Commissural Axon Guidance. Neuron 101, 625-634 e623.

-Varadarajan, S.G., Kong, J.H., Phan, K.D., Kao, T.J., Panaitof, S.C., Cardin, J., Eltzschig, H., Kania, A., Novitch, B.G., and Butler, S.J. (2017). Netrin1 Produced by Neural Progenitors, Not Floor Plate Cells, Is Required for Axon Guidance in the Spinal Cord. Neuron 94, 790-799 e793.

Thanks for pointing this out, we have replaced that reference with one of the suggestions from the referee.

4. In addition, the statement: “guidance of commissural axons does not require floor-plate-derived Netrin1", is partially correct. I find that the above Moreno-Bravo et al., paper describes best the relative contribution of the floor plate-derived Netrin.

Again, thanks for the notion, we have amended to the statement (marked in red, line 277-279).

5. Please check the spelling throughout the text to eliminate typos.

We have corrected typos (see red marked version).
