# Supplementary figures and images for: Hop Mice Display Synchronous Hindlimb Locomotion and a Ventrally Fused Lumbar Spinal Cord Caused by a Point Mutation in Ttc26
Source: eNeuro. 2022 Mar 14;9(2):ENEURO.0518-21.2022. doi: 10.1523/ENEURO.0518-21.2022 (PMC8925726; doi:10.1523/ENEURO.0518-21.2022)

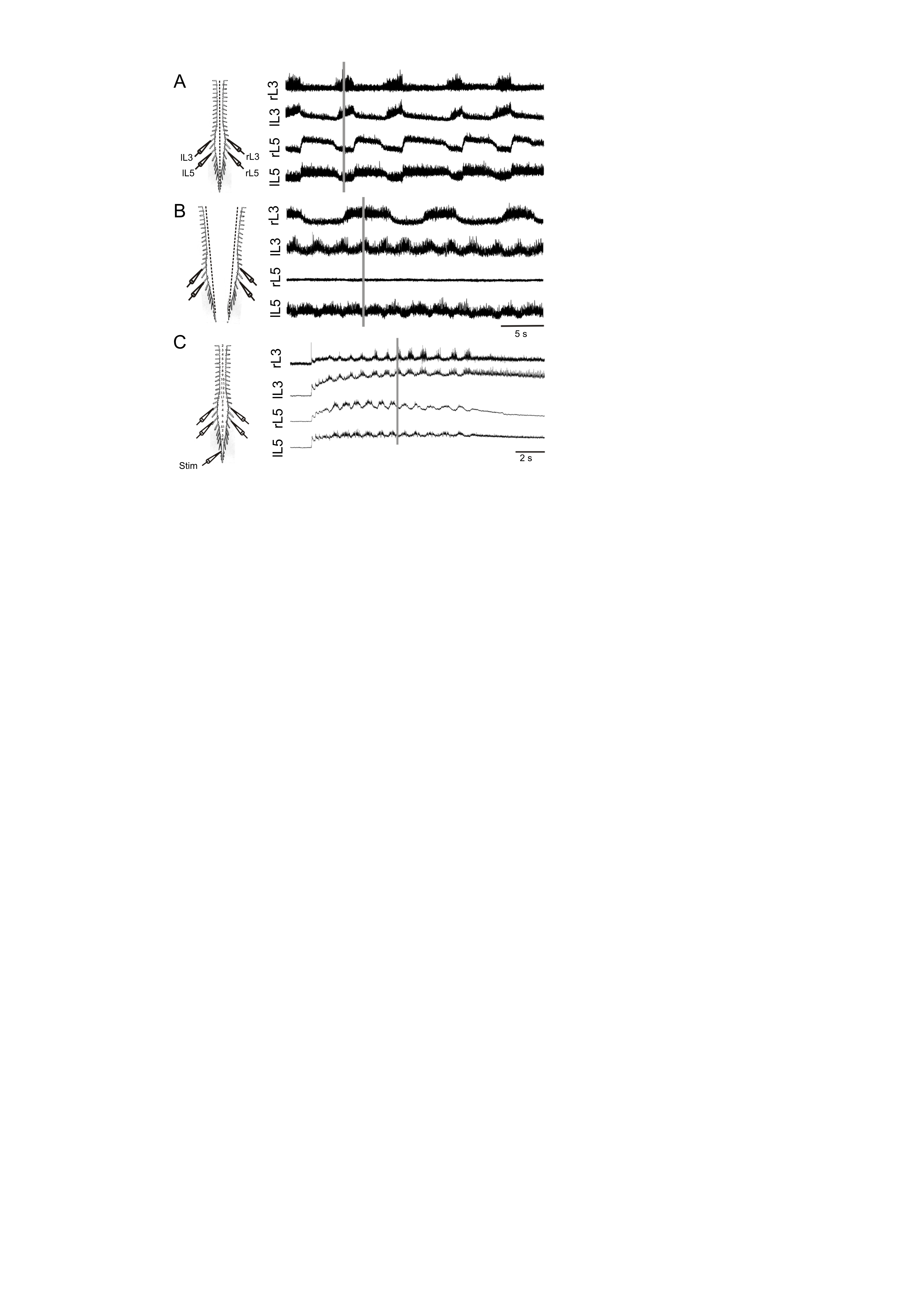

Supplement: Extended Data Figure 1-1 — Mid-sagittal hemisection does not disrupt the ipsilateral alternating burst pattern. A, Neurograms illustrating a typical pattern recorded from a hop mouse. B, Pattern recorded from the preparation following a midsagittal hemisection. Note the preservation of the L3–L5 pattern suggesting that flexor-extensor coordination is maintained. C, Schematic illustrating stimulation and recording arrangement for cauda equina stimulation. Cauda equina evoked rhythmic pattern (4 Hz, 10-s train) showed segmental synchronous pattern and ipsilateral alternating pattern. Download Figure 1-1, TIF file. [file enu-eN-NWR-0518-21-s01.tif]

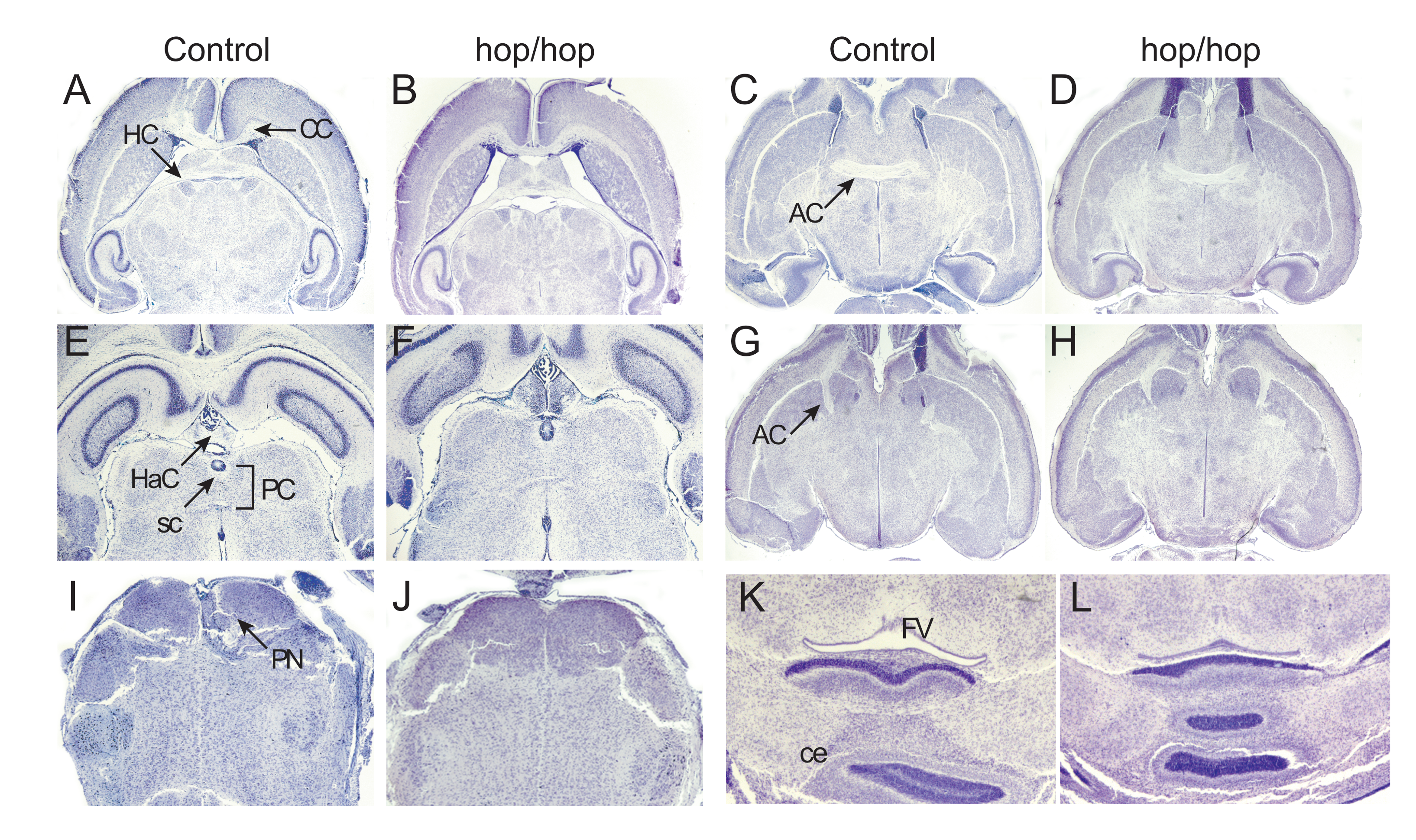

Supplement: Extended Data Figure 2-1 — Brain commissure morphology appears normal in hop mice. Photomicrographs of horizontal sections from adult control (A, C, E, G, I, K) and hop (B, D, F, H, J, L) tissue. A, B, The corpus callosum (CC) and hippocampal commissure (HC) showed no gross defects. C, D, G, H, The anterior commissure (AC) also appeared normal. E, F, Sections showing the habenular commissure (HaC), the posterior commissure (PC), and the subcommissural organ (sc), all three present and with normal appearance in both control and hop mice. I, J, The pontine nuclei (pn) is also present in both genotypes. K, L, No aberrant commissure was found between the roof of the fourth ventricle (FV), and cerebellum (ce) at the junction of midbrain and hindbrain, in control and hop mice. Scale bars: 1000 μm (A–D, G, H) and 500 μm (E, F, I–L) Download Figure 2-1, TIF file. [file enu-eN-NWR-0518-21-s02.tif]

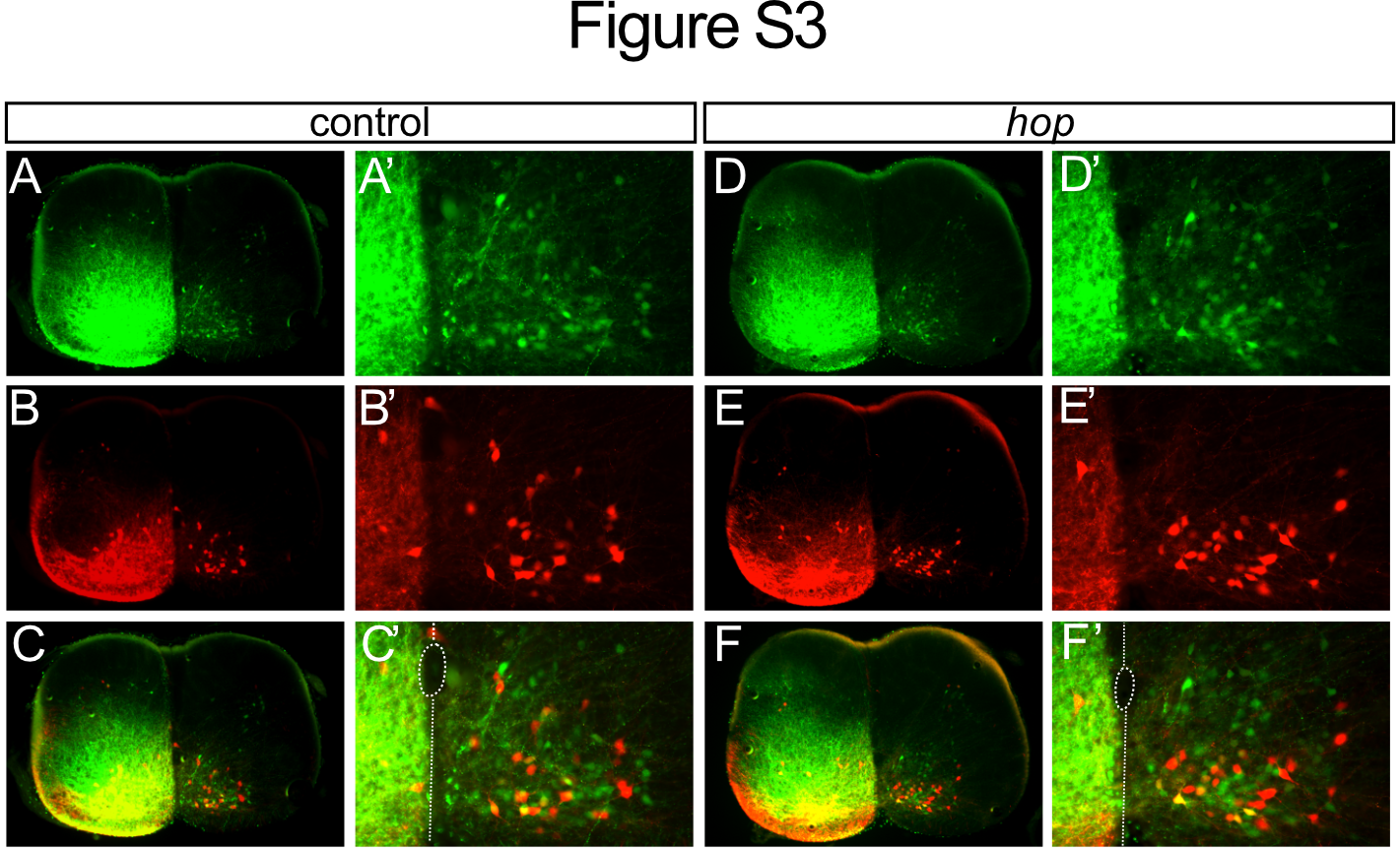

Supplement: Extended Data Figure 4-1 — Normal CINs and axon midline crossing in the cervical spinal cord of early postnatal (P0–P1) hop mice. A–F, Intersegmental tracing using FDA/RDA to locate ascending (aCINs), descending (dCINs), and bifurcating CINs (adCINs). No apparent defects in hop mice (D–F') compared to controls (A–C') on cervical sections were found. Download Figure 4-1, TIF file. [file enu-eN-NWR-0518-21-s03.tif]

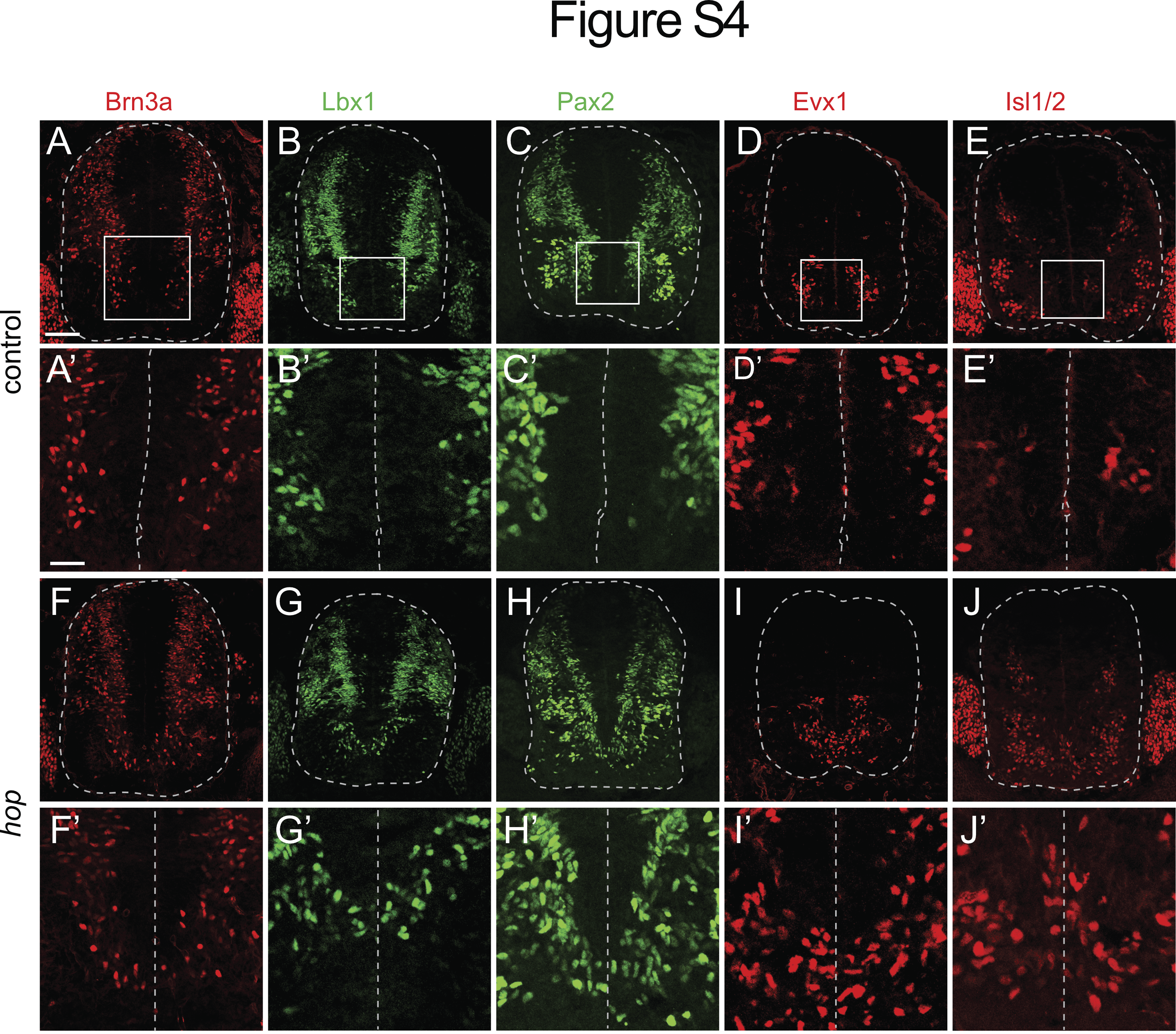

Supplement: Extended Data Figure 6-1 — Embryonic patterning defects in hop spinal cords. Photomicrographs of immunohistochemistry experiments on E12.5 spinal cord transverse sections using antibodies against transcription factors as indicated at the top. White boxes in A–E indicate areas of higher magnification in the respective panel below (A'–E'). The stainings show misplaced cells and lack of ventral midline for all tested progenitor markers. The outline of the spinal cord, central canal, and midline are indicated by dashed lines. Scale bars: 100 μm (A–J), 50 μm (A', F'), and 30 μm (B', G'–J'). Download Figure 6-1, TIF file. [file enu-eN-NWR-0518-21-s04.tif]

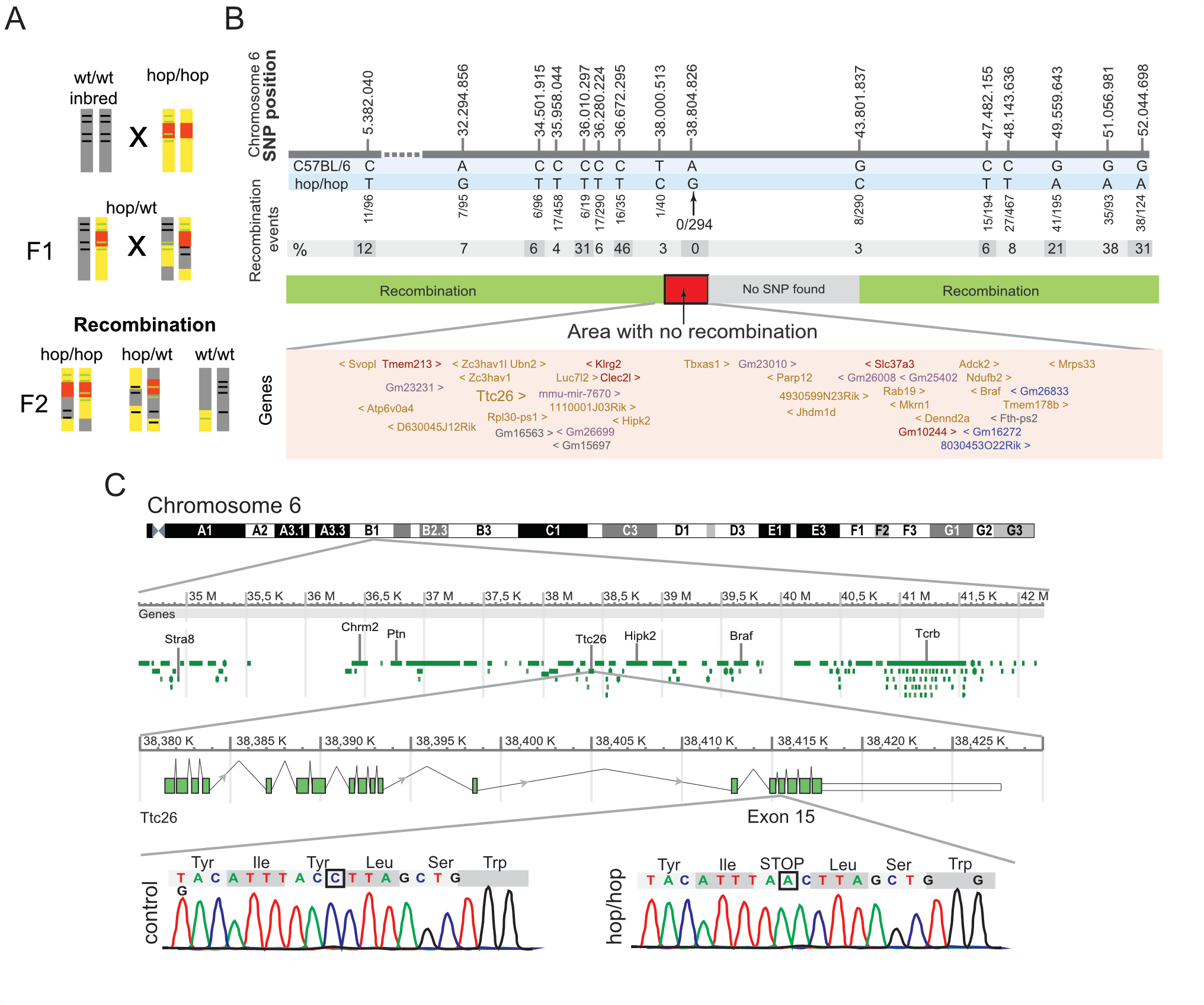

Supplement: Supplementary file 5 — Extended Data Figure 7-1. Validation of the hop mouse mutation. The exon/intron organization of Ttc26 is indicated together with Sanger sequencing traces of wild type and hop highlighting the premature stop mutation in exon 15 (out of 18) of Ttc26. Genomic DNA from a hop homozygote (stock 002718) and BALB/cByJ control (stock 001026) was obtained from The Jackson Laboratory’s DNA Resource (n = 3 per genotype). Exomes were captured at the Yale Center for Genome Analysis, using the NimbleGen SeqCap EZ Mouse Exome and following NimbleGen protocols. Captured pools were sequenced (75 bp, paired-end) on an Illumina HiSeq 2000 using previously described methods (Choi et al., 2009). We obtained ∼91 million (BALB/cByJ) to ∼113 million (hop) high-quality reads. Illumina reads were first trimmed based on their quality scores to remove low-quality regions using the program Btrim (Kong, 2011). A cutoff of 20 for average quality scores within a moving window of size 5-bp was used. Minimum acceptable read length was 25 bp. Other parameters of Btrim were set to defaults. The preprocessed reads were then aligned to the mouse genome reference sequence (mm9) using the BWA mapping program (Li and Durbin, 2009). The mapping results were converted into SAMtools pileup format using SAMtools programs (Li et al., 2009). PCR duplicates were removed using the rmdup command from SAMtools, resulting in ∼84x (BALB/cByJ) or ∼100x (hop) coverage across the exome. More than 97% of all bases included in the exome showed at least 8x coverage and >90% of the bases showed at least 20x coverage. Single nucleotide variations (SNVs) were called using SAMtools’ pileup command. Further filtering was performed using in-house scripts to exclude those SNV calls that had less than 3 reads or a SNP score less than 20. Annotation was added based on the UCSC RefSeq gene model (http://genome.ucsc.edu/; Pruitt et al., 2009). Based on SNV homozygosity mapping, the interval was narrowed to ∼700 kb on chromosome 6 (37.9 [file enu-eN-NWR-0518-21-s05.tif]

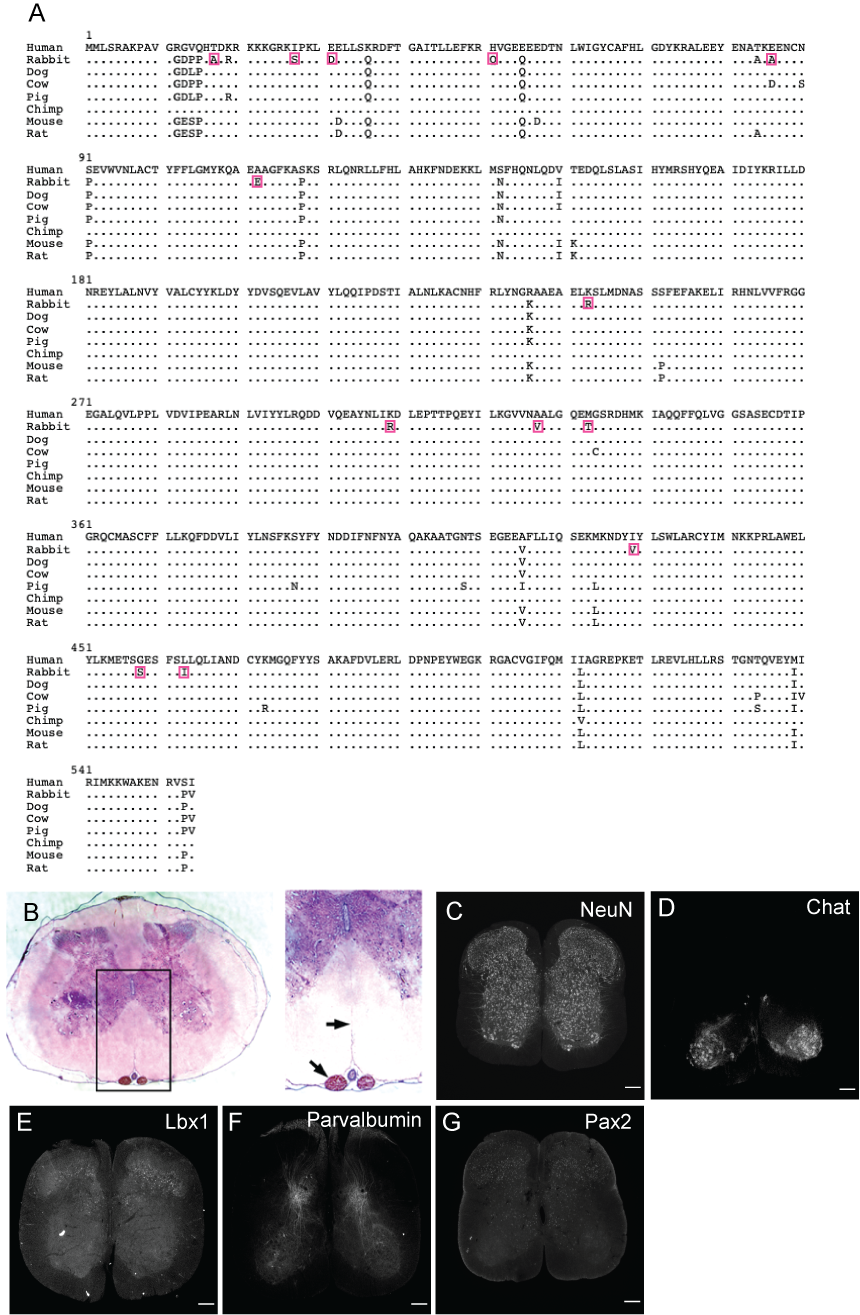

Supplement: Extended Data Figure 7-2 — Relatively high number of missense mutations in the rabbit Ttc26 gene. A, Amino acid alignment of TTC26 sequences from eight mammals made using MUSCLE with missense mutations in rabbit marked (pink boxes). B, Photomicrograph of HE stained lumbar spinal cord section from wild-type adult rabbit (box shown magnified region). The morphology is similar to the wild-type mouse spinal cord, with a well delineated central canal (upper arrow), a ventral funiculus with a clear border between white and grey matter and a ventral spinal artery (lower arrow). C–G, Immunohistochemistry performed on postnatal rabbits towards NeuN (C), Chat (D), Lbx1 (E), Parvalbumin (F), and Pax2 (G) show a clear bilateral separation of the spinal neuronal populations. Scale bars: 200 μm (C–G). Download Figure 7-2, TIF file. [file enu-eN-NWR-0518-21-s06.tif]
